# Healthcare utilization and clinical characteristics of genetic epilepsy in electronic health records

**DOI:** 10.1093/braincomms/fcae090

**Published:** 2024-03-14

**Authors:** Christian M Boßelmann, Alina Ivaniuk, Mark St John, Sara C Taylor, Gokul Krishnaswamy, Alex Milinovich, Costin Leu, Ajay Gupta, Elia M Pestana-Knight, Imad Najm, Dennis Lal

**Affiliations:** Genomic Medicine Institute, Lerner Research Institute, Cleveland Clinic, Cleveland, OH 44195, USA; Epilepsy Center, Neurological Institute, Cleveland Clinic, Cleveland, OH 44195, USA; Genomic Medicine Institute, Lerner Research Institute, Cleveland Clinic, Cleveland, OH 44195, USA; Epilepsy Center, Neurological Institute, Cleveland Clinic, Cleveland, OH 44195, USA; Genomic Medicine Institute, Lerner Research Institute, Cleveland Clinic, Cleveland, OH 44195, USA; Epilepsy Center, Neurological Institute, Cleveland Clinic, Cleveland, OH 44195, USA; Neurological Institute, Cleveland Clinic, Cleveland, OH 44195, USA; Neurological Institute, Cleveland Clinic, Cleveland, OH 44195, USA; Department of Quantitative Health Sciences, Lerner Research Institute, Cleveland Clinic, Cleveland, OH 44195, USA; Genomic Medicine Institute, Lerner Research Institute, Cleveland Clinic, Cleveland, OH 44195, USA; Department of Clinical and Experimental Epilepsy, Institute of Neurology, University College London, London, WC1N 3BG, UK; Department of Neurology, The University of Texas Health Science Center at Houston, Houston, TX 77030, USA; Center for Neurogenetics, The University of Texas Health Science Center at Houston, Houston, TX 77030, USA; Epilepsy Center, Neurological Institute, Cleveland Clinic, Cleveland, OH 44195, USA; Epilepsy Center, Neurological Institute, Cleveland Clinic, Cleveland, OH 44195, USA; Epilepsy Center, Neurological Institute, Cleveland Clinic, Cleveland, OH 44195, USA; Genomic Medicine Institute, Lerner Research Institute, Cleveland Clinic, Cleveland, OH 44195, USA; Epilepsy Center, Neurological Institute, Cleveland Clinic, Cleveland, OH 44195, USA; Department of Neurology, The University of Texas Health Science Center at Houston, Houston, TX 77030, USA; Center for Neurogenetics, The University of Texas Health Science Center at Houston, Houston, TX 77030, USA; Stanley Center for Psychiatric Research, Broad Institute of Harvard and M.I.T., Cambridge, MA 02142, USA; Cologne Center for Genomics (CCG), University of Cologne, 50931 Cologne, Germany

**Keywords:** electronic health record, genetics, epilepsy, phenotyping

## Abstract

Understanding the clinical characteristics and medical treatment of individuals affected by genetic epilepsies is instrumental in guiding selection for genetic testing, defining the phenotype range of these rare disorders, optimizing patient care pathways and pinpointing unaddressed medical need by quantifying healthcare resource utilization. To date, a matched longitudinal cohort study encompassing the entire spectrum of clinical characteristics and medical treatment from childhood through adolescence has not been performed. We identified individuals with genetic and non-genetic epilepsies and onset at ages 0–5 years by linkage across the Cleveland Clinic Health System. We used natural language processing to extract medical terms and procedures from longitudinal electronic health records and tested for cross-sectional and temporal associations with genetic epilepsy. We implemented a two-stage design: in the discovery cohort, individuals were stratified as being ‘likely genetic’ or ‘non-genetic’ by a natural language processing algorithm, and controls did not receive genetic testing. The validation cohort consisted of cases with genetic epilepsy confirmed by manual chart review and an independent set of controls who received negative genetic testing. The discovery and validation cohorts consisted of 503 and 344 individuals with genetic epilepsy and matched controls, respectively. The median age at the first encounter was 0.1 years and 7.9 years at the last encounter, and the mean duration of follow-up was 8.2 years. We extracted 188,295 Unified Medical Language System annotations for statistical analysis across 9659 encounters. Individuals with genetic epilepsy received an earlier epilepsy diagnosis and had more frequent and complex encounters with the healthcare system. Notably, the highest enrichment of encounters compared with the non-genetic groups was found during the transition from paediatric to adult care. Our computational approach could validate established comorbidities of genetic epilepsies, such as behavioural abnormality and intellectual disability. We also revealed novel associations for genitourinary abnormalities (odds ratio 1.91, 95% confidence interval: 1.66–2.20, *P* = 6.16 × 10^−19^) linked to a spectrum of underrecognized epilepsy-associated genetic disorders. This case-control study leveraged real-world data to identify novel features associated with the likelihood of a genetic aetiology and quantified the healthcare utilization of genetic epilepsies compared with matched controls. Our results strongly recommend early genetic testing to stratify individuals into specialized care paths, thus improving the clinical management of people with genetic epilepsies.

## Teaser

Boßelmann *et al*. demonstrate how natural language processing of electronic health records can be used to identify individuals with genetic epilepsy. These individuals had earlier diagnoses and more complex healthcare encounters, especially during transition from paediatric to adult care.

## Graphical Abstract

**Graphical Abstract:**
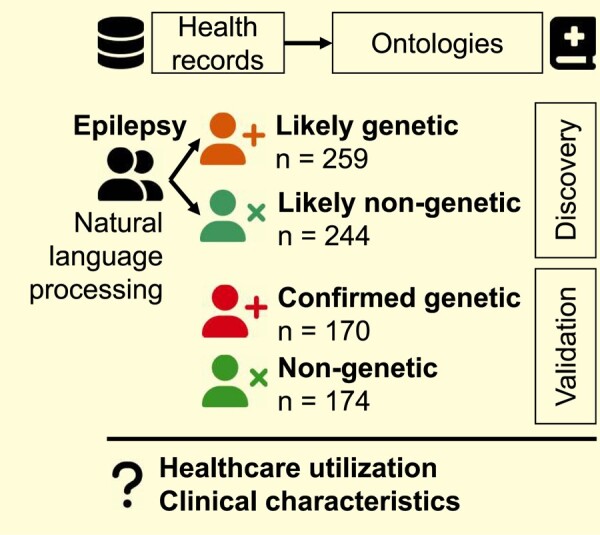

## Introduction

Many forms of epilepsy are likely to have a genetic aetiology, ranging from rare *de novo* monogenic syndromes like developmental and epileptic encephalopathies (DEE) to polygenic burden in common focal and generalized epilepsies.^1,2^ Overall, >140 epilepsy-associated genes have been identified.^3^ While individually rare, the annual incidence of genetic epilepsies is estimated to be 1 per 2120 live births.^3^ Many of the monogenic epilepsies now have therapeutic and prognostic implications.^4-7^ Thus, genetic testing is vital to address the unmet medical needs of these often severely affected individuals, as it facilitates a timely diagnosis, informs clinical management and ultimately enables candidate precision therapies or clinical trial readiness.^8^ Certain clinical characteristics increase the pre-test probability of an underlying genetic aetiology and thus improve the diagnostic yield of genetic testing.^9^ Hence, the Genetics Commission of the International League Against Epilepsy (ILAE) currently recommends genetic testing in cases with certain additional symptoms, including intellectual disability, autism and dysmorphology.^10^ Identifying clinical characteristics that are independently associated with a genetic aetiology may therefore improve patient selection for testing.

The clinical characteristics of genetic epilepsies were historically defined by careful observation of small cohorts. More recently, electronic health records (EHR) have been applied to scale this discovery process to the large amount of data available today. Standardized vocabularies, annotation pipelines and ontological reasoning have enabled this approach and partially addressed the inherent limitations of using large-scale real-world data.^11,12^ Deep quantitative phenotypic analysis has greatly enhanced our understanding of the clinical spectrum of disorders related to variants in *SCN2A*,^13^  *STXBP1*^14^ and others. Longitudinal approaches have examined the disease trajectories of rare disorders to identify age-dependent patterns in their clinical features across thousands of patient years.^15,16^ While previous work has focused on deep data analysis from individuals with variants in known epilepsy-related genes, the practical implications for a larger and more general population sample remain unclear. Individuals with childhood-onset genetic epilepsies are known to have heterogeneous clinical features^17^ and are affected by high rates of psychiatric and somatic comorbidities.^18^ Their disease progression from childhood to adolescence and the impact on healthcare resource utilization and medical treatment remain poorly understood.

Here, we conducted a case-control cross-sectional and longitudinal study on EHR data from individuals with known or likely genetic epilepsy against matched controls with epilepsy across a large healthcare network. We set out to describe the disease progression, comorbidities and medical treatment of individuals with genetic epilepsies. Our data-driven whole-phenome approach identifies novel clinical features predictive of a genetic aetiology and highlights the unmet medical needs of these individuals.

## Materials and methods

### Setting and participants

This study was carried out at the main campus and 14 northeastern Ohio affiliate hospitals of the Cleveland Clinic Health System of the Cleveland Clinic Foundation. EHR were queried for entries between 1 January 1998 and 31 January 2023. The study site is a Level 4 Adult and Paediatric Epilepsy Centre accredited by the National Association of Epilepsy Centers (NAEC). We chose the setting of a large healthcare system network to reduce the impact of single providers, enable data sharing across sites and benefit from standardized professional guidelines and coding practices. All sites used Epic electronic medical records (Epic Systems Corporation, WI, USA).

Eligibility criteria to identify epilepsy cases were (i) any International Classification of Diseases, Tenth Revision, Clinical Modification (ICD-10-CM) code G40- (‘Epilepsy and recurrent seizures’) or ICD-9 code 345.*, (ii) any Current Procedural Terminology (CPT) code for EEG and (iii) age 0–5 years at the time of diagnosis (first billing code for epilepsy). Eligibility criteria were based on a systematic meta-review on the accuracy of using administrative healthcare data to identify epilepsy cases, where the positive predictive value and sensitivity of nine validation studies in the USA ranged from 32.7% to 96.0% and 12.2% to 97.3%, respectively.^19^ We chose strict cohort definitions based on two rationales: (i) participants who had received CPT codes for EEG may be more likely to have been diagnosed within the healthcare system, increasing length and depth of follow-up, and (ii) participants should be strongly enriched for epilepsy while removing those with unclear diagnoses such as convulsions or syncope (i.e. high precision at the cost of sensitivity).

For the discovery cohort, participants were then stratified into case-control groups: likely genetic individuals had ≥1 order for any genetic testing and a match for a custom natural language processing (NLP) algorithm (Supplementary Table 1). For the validation cohort, we conducted manual chart review of each case and established if the individual had a confirmed genetic diagnosis, i.e. a finding of at least one variant in a known disease-related gene that was classified as (likely) pathogenic according to American College of Medical Genetics and Genomics (ACMG) criteria.^20^ The age at genetic testing or diagnosis of a genetic disorder was not restricted to account for potential diagnostic delays. This strict subset of cases was matched to an independent set of controls that had ≥1 order for any genetic testing and a negative match on our custom NLP algorithm. Additional individuals that fulfilled the eligibility criteria were identified by ICD-10 codes for monogenic syndromes, including tuberous sclerosis complex (ICD-10 85.1, *n* = 17), cyclin-dependent kinase-like 5 deficiency disorder (CDKL5-DD, ICD-10 G40.42, *n* = 11) or Dravet syndrome (ICD-10 G40.834, *n* = 13). For additional validation, we implemented PheIndex, a recently developed algorithm to identify individuals with rare genetic disorders and found a strong correlation with our group labels (Supplementary Fig. 1).^21^

For case-control matching, we applied three matching criteria with the following rationales: (i) sex, as several genetic epilepsies and their comorbidities have sex-dependent phenotypic features; (ii) median age, to control for differences in age-dependent longitudinal phenotypes and changes in billing or coding practices; and (iii) self-reported ancestry, to minimize systematic bias of our genetic risk estimates by ancestry-dependent population substructure. Matching was done by propensity score matching with scores estimated by a generalized linear model followed by nearest-neighbour matching at the default 1:1 ratio.^22^ After matching, the discovery cohort consisted of 503 individuals and the validation cohort consisted of 344 individuals. Due to the nature of the retrospective EHR-based study design, information on individuals lost to follow-up was unavailable.

We note that idiopathic (genetic) generalized epilepsy (IGE) was not the focus of this study, due to the more nuanced phenotypes and complex (polygenic) genetics. These cases were not manually removed from the cohorts in order to avoid introducing further bias and because some IGE with comorbidities (e.g. autism) may have a higher pre-test probability of being genetic.

### Variables

**Figure 1:**
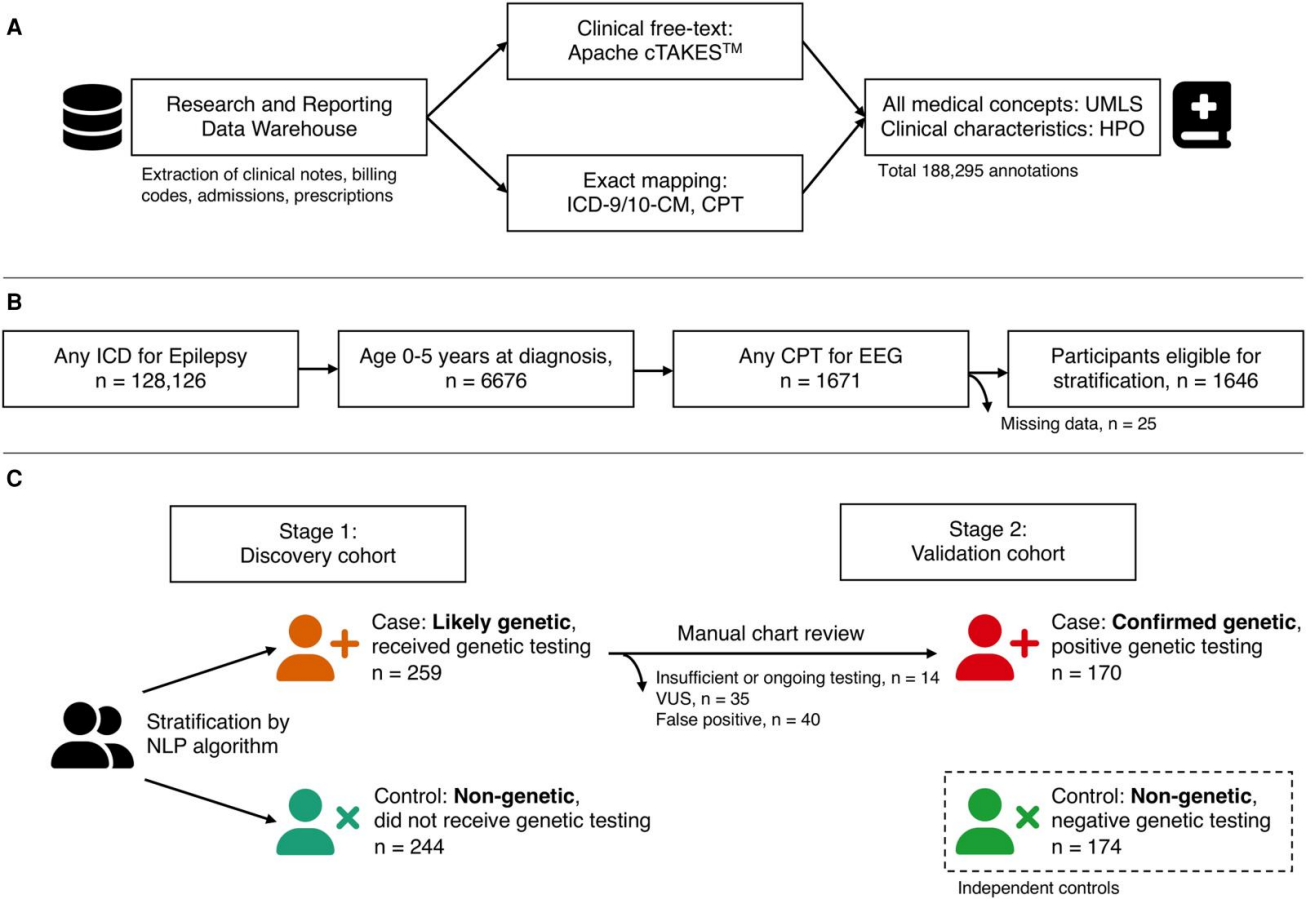
**Flow diagram of data sets and processes used in the study.** (**A**) Data extraction and annotation pipeline. (**B**) Patient selection diagram. (**C**) Study design overview. CPT, Current Procedural Terminology; EEG, electroencephalography; EHR, electronic health records; HPO, Human Phenotype Ontology; ICD-9/10-CM, International Classification of Disease, Clinical Modification; NLP, natural language processing; UMLS, Unified Medical Language System; VUS, variant of unknown significance.

The investigators had full access to the database population used to create the study population. Data set construction, cleaning and person-level linkage across databases (Fig. 1) were carried out as previously described.^11^ The Research Data Warehouse at the Cleveland Clinic is an in-house relational database that maps Unified Medical Language System (UMLS, release 2022AA) concepts to integrate and standardize clinical data. This process includes automatic source code matching (2011 ICD-9-CM, 2023 ICD-10-CM, CPT 2021), exact or fuzzy text matching to raw clinical notes (Apache cTAKES™) and manual mapping. More than 70% of data is mapped automatically, and the system has been previously validated across a wide range of use cases.^11^ This procedure resulted in a list of UMLS concept annotations for each person with epilepsy at every encounter. Duplicates were removed, and concepts were grouped if their encounters occurred within 1 month of each other (as codes generated during billing, lab results or late documentation were assigned different dates). These concepts were then mapped to Human Phenotype Ontology (HPO, v2023–01–27) terms, a standardized vocabulary of phenotypic features.^12^ The use of the HPO as a phenotyping algorithm has been previously validated, and the process of propagating sets of terms to enable ontological reasoning has been previously described (Supplementary Fig. 2).^13,14^ The comprehensive ontological system of the UMLS and HPO reduces potential bias by standardizing variable definitions and removing the need for feature selection in favour of a hypothesis-free approach.

After stratification, 25 individuals were removed due to missing data in encounter date annotations which could not be confirmed as missing at random. No missing data imputation was done. Quantitative variables included age at the encounter, age at the last follow-up and age at diagnosis. For longitudinal analyses, we grouped these according to the age ranges used by the ILAE Task Force on Nosology and Definitions: 0–2 years (neonatal/infantile), 2–12 years (childhood), 12–18 years (juvenile) and >18 years (adult).^23^

This study is reported according to the STROBE-RECORD extended checklist and meets all five CODE-EHR minimum best practice framework standards for using structured healthcare data in clinical research.^24^

### Statistical analysis

This study was conducted in the R programming language, version 4.1.0, with RStudio, version 1.4.1106. We used two-sided Fisher’s exact or *t*-tests to test for association between variables and genetic aetiology, where appropriate. The threshold for statistical significance was set to α = 0.05. *P*-values were adjusted for multiple testing with Bonferroni’s correction for whole-phenome analyses (i.e. association testing across all UMLS concepts or all HPO terms), and corrected *P*-values (*P*_adj_) are reported where appropriate. The effect sizes of relative enrichment were provided as odds ratios (ORs) with 95% confidence intervals (CIs).

### Ethics statement

This study was approved by the Institutional Review Board of the Cleveland Clinic, approval IDs #22–147 and #23–253. Informed consent was waived due to the retrospective study design. All concept associations were deidentified to ensure data privacy, and all data were processed and stored on secure infrastructure.

## Results

### Healthcare utilization is higher in individuals with genetic epilepsy

**Figure 2:**
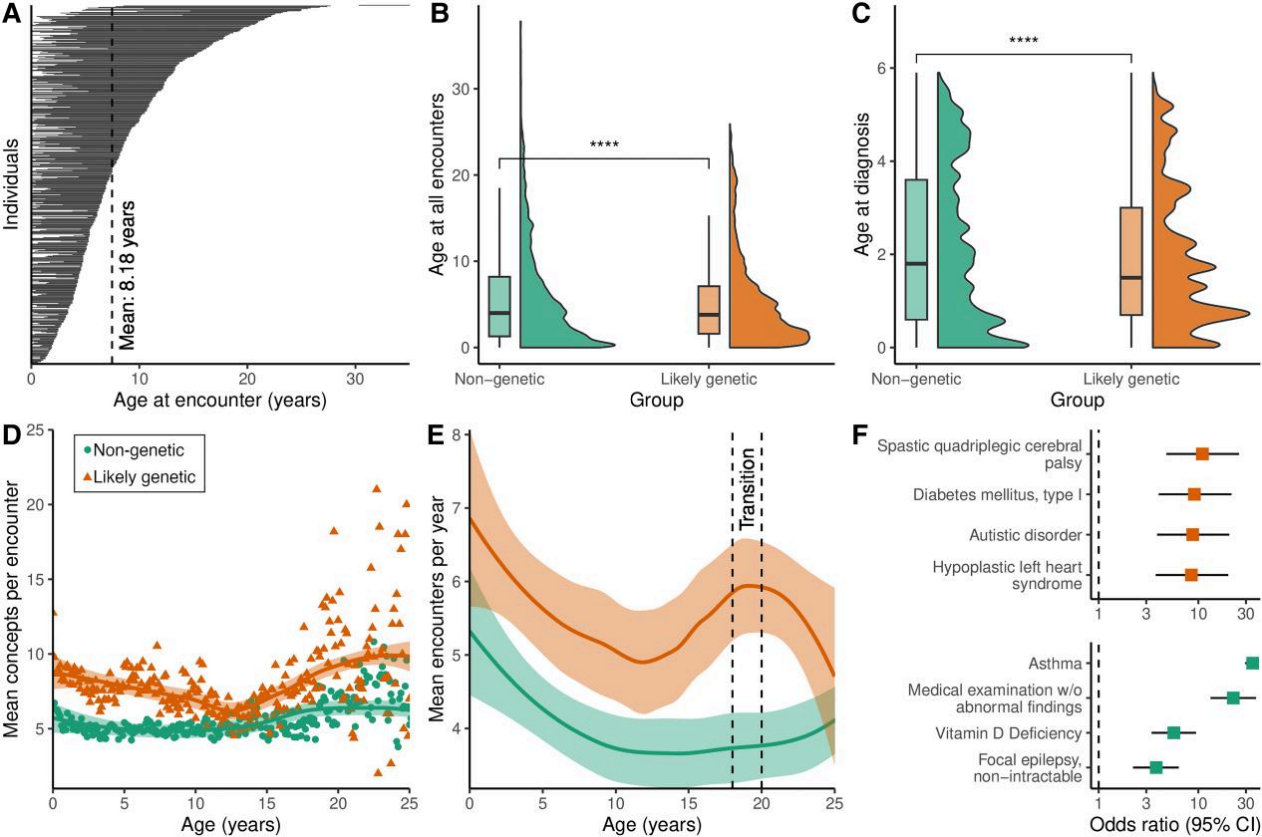
**Length of follow-up, age distribution and encounter distribution for the discovery cohort.** (**A**) Length of follow-up for each individual (*n* = 504) is shown as stacked horizontal lines, sorted by age at the last follow-up. Each line represents the length of EHR data available. (**B**) Violin and boxplot of age in years at all encounters for individuals with likely genetic and non-genetic epilepsies. Two-sided *t*-test, ****P < 0.0001, *t* = 38.74. (**C**) Violin and boxplot of age in years at diagnosis (fulfilment of eligibility criteria) for individuals with likely genetic and non-genetic epilepsy. Two-sided *t*-test, ****P < 0.0001, *t* = 53.66. (**D**) Mean number of UMLS concepts per encounter for each group. Each dot is the mean number of monthly concepts per group. (**E**) Mean number of annual encounters per year for each group (likely genetic, *n* = 259 and non-genetic, *n* = 244). The line corresponds to the smooth conditional mean, with the shaded area being the standard error of the mean. The dashed lines mark the largest relative difference in annual encounter frequency, the transition period from paediatric to adult care (ages 18–20 years). (**F**) Top four UMLS concepts with the greatest enrichment in the transition period. Forest plot of concept enrichment during the transition period compared with before the transition period, sorted by highest OR and shown separately for each group.

Genetic epilepsies are likely to have different healthcare utilization patterns that have not yet been quantified in a controlled study. Here, we included participants with childhood-onset epilepsy, where individuals with genetic epilepsy were identified by NLP. The discovery cohort consisted of 259 individuals with known or likely genetic epilepsy and 244 matched controls (Table 1). Their ICD-10 diagnoses are shown in Supplementary Table 2. The mean length of follow-up was 8.18 years (median 7, SD 5.01, range 0.10–21.70) for a cumulative follow-up of 4115 person-years (Fig. 2A), and each individual had an average of 19.20 encounters within the healthcare system (median 11, SD 21.10, range 1–144). The median age at the first and last encounter was 0.1 years and 7.9 years, respectively. EHR extraction yielded a total of 188,295 annotations across 9659 encounters, with a mean of 8.94 unique UMLS concepts (SD 8.62, range 1–54) and 19.2 HPO terms (SD 21.1, range 1–144) per individual. Each annotation corresponded to one single diagnostic or procedural concept mapped from raw text in clinical notes, billing information or diagnostic results.

**Table 1:** Demographic features of the discovery and validation cohorts

Discovery cohort				
Variable		Likely genetic	Non-genetic (no genetic testing)	*P*-value
Sex	Female, *n* (%)	117 (45.2)	114 (46.7)	0.796
	Male, *n* (%)	142 (54.8)	130 (53.3)	
Ancestry	Hispanic or Latino, *n* (%)	20 (7.7)	20 (8.2)	0.420
	Not Hispanic or Latino, *n* (%)	227 (87.6)	218 (89.3)	
	Unknown, *n* (%)	12 (4.6)	6 (2.5)	
Age	Years, median (SD)	5.3 (5.2)	5.2 (4.8)	0.711

Validation cohort				
Variable		Confirmed genetic	Non-genetic (negative genetic testing)	*P*-value
Sex	Female, *n* (%)	84 (49.4)	81 (46.6)	0.672
	Male, *n* (%)	86 (50.6)	93 (53.4)	
Ancestry	Hispanic or Latino, *n* (%)	13 (7.6)	8 (4.6)	0.306
	Not Hispanic or Latino, *n* (%)	152 (89.4)	157 (90.2)	
	Unknown, *n* (%)	5 (2.9)	9 (5.2)	
Age	Years, median (SD)	5.3 (4.7)	6.2 (5.2)	0.112

Individuals with likely genetic epilepsy were younger when they had any healthcare encounters (mean age 5.29 years versus 5.83, two-sided *t*-test, *P* = 4.9 × 10^−7^; Fig. 2B) and were younger when they were first diagnosed with epilepsy (age at ICD-10 G40.-, mean age 1.87 years versus 2.09, two-sided *t*-test, *P* < 2.22 × 10^−16^; Fig. 2C). Over the entire age range, individuals with likely genetic epilepsy received more annotations per encounter [mean concepts per encounter 8.13 (SD = 2.72) versus 5.90 (SD = 2.71), two-sided *t*-test, *P* = 5.81 × 10^−22^] (Fig. 2D), as a surrogate marker for phenotypic complexity or healthcare utilization. Out of the 354/503 (70%) of individuals admitted to the emergency department at least once, likely genetic individuals were admitted significantly more often [mean admissions 20.00 (SD = 16.30) versus 12.30 (SD = 9.40), two-sided *t*-test, *P* = 1.06 × 10^−46^]. Likewise, out of the 258/503 (51%) of individuals admitted to the inpatient service at least once, likely genetic individuals were significantly more likely to be admitted more often [mean admissions 12.40 (SD = 15.30) versus 8.55 (SD = 8.78), two-sided *t*-test, *P* = 0.009].

Healthcare resource utilization may vary over time, and individuals with genetic epilepsy are known to require multidisciplinary care during the transition from paediatric to adult care.^25^ Indeed, the largest relative increase in annual encounters compared with controls was seen at ages 18–20 years [mean annual encounters 6.17 (SD = 3.26) versus 3.63 (SD = 2.63), two-sided *t*-test, *P* = 3.3 × 10^−7^] (Fig. 2E). Compared with encounters before transition, encounters in likely genetic individuals during the transition were enriched for cerebral palsy, autistic disorder or severe somatic comorbidities. Encounters of non-genetic individuals were enriched for asthma, medical examinations without abnormal findings or non-intractable epilepsy (Fig. 2F).

### Individuals with genetic epilepsy have a distinct spectrum of associated clinical features

**Figure 3:**
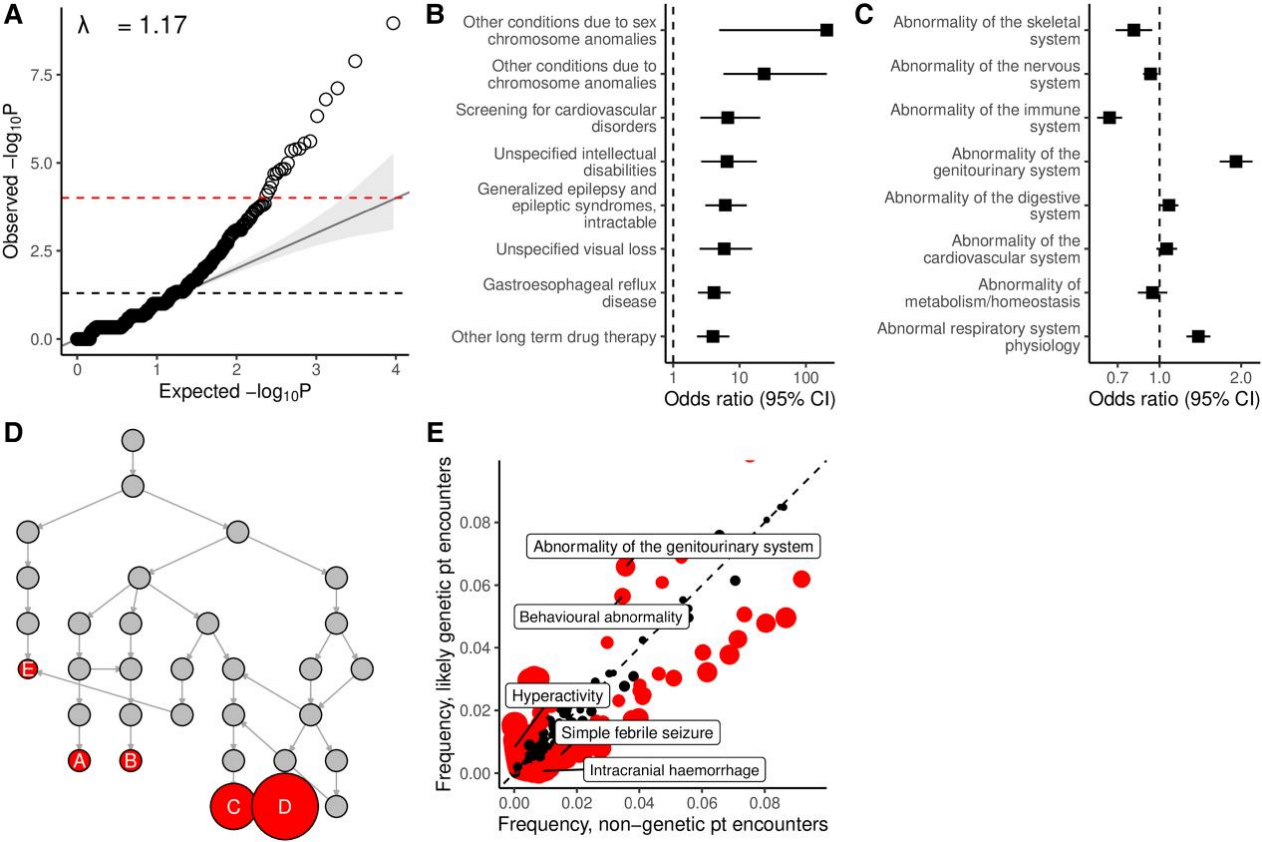
**Cross-sectional analysis of clinical features associated with a likely genetic aetiology. (A)** Quantile–quantile (QQ) plot of the −log_10_ scaled nominal observed versus expected *P*-value distribution for all tested hypotheses (*n* = 512; UMLS concept association), showing minimal *P*-value inflation (λ=1.17). The nominal significance threshold (α=0.05) and Bonferroni-corrected significance threshold (*α* = 9.67 × 10^−5^) are shown as dashed lines. (**B**) Forest plot of the top 10 UMLS concepts most enriched in individuals with likely genetic epilepsies, sorted by OR. (**C**) Forest plot of system-level HPO terms that are children of phenotypic abnormality (HP:0000118). (**D**) Reingold–Tilford tree visualization of the subgraph rooted at abnormality of the genitourinary system (HP:0000119). Nodes shown with labels are terms that are independently significantly associated with individuals with likely genetic epilepsy and are labelled by the term they represent: A, renal cyst (HP:0000107); B, chronic kidney disease (HP:0012622); C, penile hypospadias (HP:0003244); D, cryptorchidism (HP:0000028); and E, urinary incontinence (HP:0000020). (**E**) Relative frequency of HPO terms (*n* = 1137) in encounters for individuals with likely genetic epilepsies versus those with non-genetic epilepsy. Each dot corresponds to a single term and is coloured red if significant.

Individuals with likely genetic epilepsy may have distinct clinical features compared with controls with non-genetic epilepsy. We, therefore, extracted 188,295 annotations across 9659 encounters from the EHR and established cross-sectional phenotypes by comparing the presence or absence of any of the >900 000 UMLS concepts and >13 000 HPO terms, with each hypothesis corrected for multiple testing. We report adjusted *P*-values (*P*_adj_) throughout this section. Test statistics showed only minimal *P*-value inflation (λ=1.17; Fig. 3A). UMLS concepts were used to reflect general diagnostics, as billing and procedural information may not directly map to phenotypic features represented in the HPO. Likely genetic individuals were enriched for UMLS concepts including chromosomal anomalies, intractable generalized epilepsy and intellectual disability (Fig. 3B). We used HPO terms to complement UMLS concepts for more detailed analyses across the entire clinical spectrum.

We grouped annotations by system-level terms and noted that likely genetic individuals were enriched for abnormalities of the genitourinary system, a novel finding with a moderate effect size (HP:0000119; OR 1.91, 95% CI: 1.66–2.20, *P*_adj_ = 6.16 × 10^−19^; Fig. 3C). Several clinical features contributed to this signal and were independently associated with likely genetic individuals: cryptorchidism (HP:0000028, *P*_adj_ = 2.62 × 10^−25^), penile hypospadias (HP:0003244, *P*_adj_ = 1.67 × 10^−15^), chronic kidney disease (HP:0012622, *P*_adj_ = 1.10 × 10^−7^) and others (Fig. 3D). More fine-grained phenotypic representations are shown in Fig. 3E, where we found likely genetic individuals to be enriched for behavioural abnormality (HP:0000708, OR 1.66, 95% CI: 1.45–1.90, *P*_adj_ = 5.64 × 10^−11^), including hyperactivity (HP:0000752, OR 24.71, 95% CI: 8.23–121.32, *P*_adj_ = 7.97 × 10^−18^), but depleted for simple febrile seizures (HP:0002373, OR 0.49, 95% CI: 0.45–0.70, *P*_adj_ = 2.97 × 10^−3^) and cerebral haemorrhage (HP:0001342, OR 0.02, 95% CI: 0.01–0.06, *P*_adj_ = 5.20 × 10^−46^), among others.

### Longitudinal analysis reveals age-dependent patterns in clinical features and medical treatment

**Figure 4:**
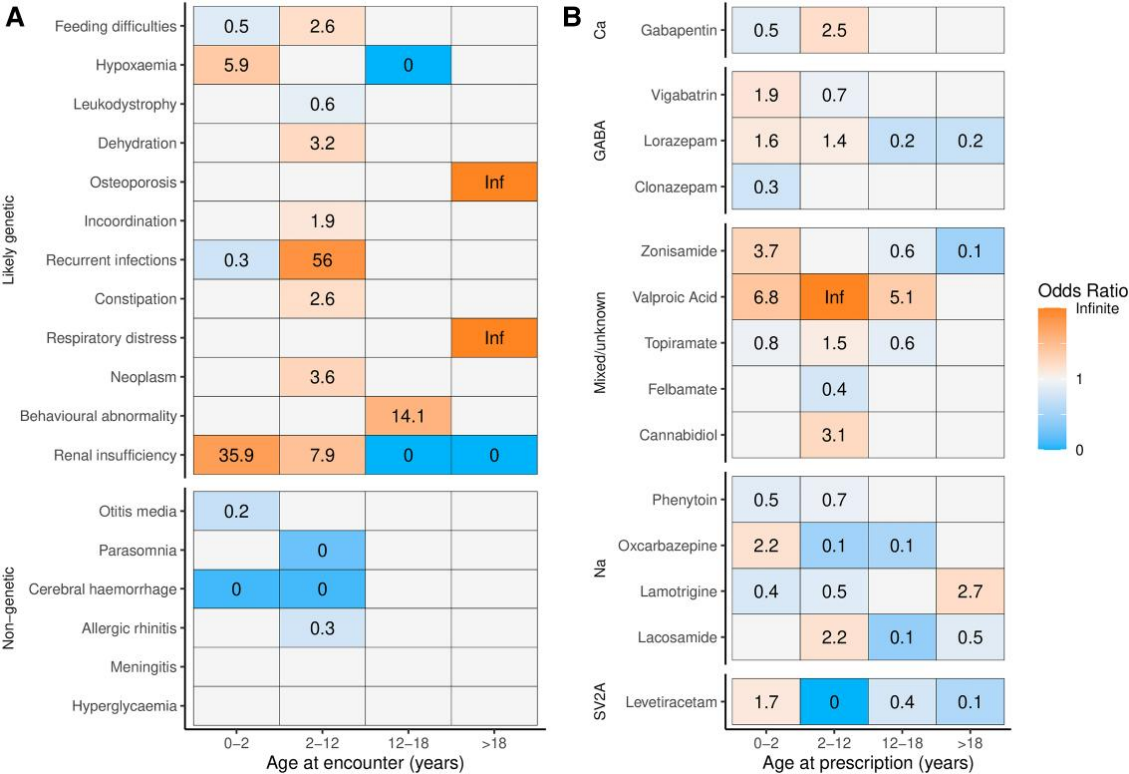
**Longitudinal analysis of clinical features associated with a likely genetic aetiology.** (**A**) Heatmap of clinical features over the age ranges, binned by neonatal/infantile (0–2 years), childhood (2–12 years), juvenile (12–18 years) and adulthood (>18 years). Relative enrichment (OR) of features between individuals with likely genetic epilepsy and those with non-genetic epilepsy is shown as labels. Blank tiles correspond to non-significant associations. Terms were grouped via hierarchical clustering of similar trajectories. (**B**) Heatmap of ASM prescription patterns, grouped by putative main mechanism of action. ASMs are shown if they had any significant group-level associations and were prescribed to at least 1% of the study cohort.

Genetic disorders associated with epilepsy are not static but represent dynamic entities with age-dependent clinical features. Identifying the timepoints where actionable phenotypes occur can inform diagnostic surveillance and clinical management. We, therefore, examined associated clinical features across age groups from infancy (0–2 years), childhood (2–12 years), youth (12–18 years) and adulthood (>18 years). Likely genetic individuals were significantly more likely to have recurrent infections (HP:0002719, OR 55.99, 95% CI: 9.87–2203.37, *P*_adj_ = 3.76 × 10^−16^), feeding difficulties (HP:0011968, OR 2.57, 95% CI: 2.09–3.18, *P*_adj_ = 4.11 × 10^−21^), constipation (HP:0002019, OR 2.58, 95% CI: 2.06–3.27, *P*_adj_ = 6.60 × 10^−17^) or dehydration (HP:0001944, OR 3.19, 95% CI: 2.19–4.81, *P*_adj_ = 3.69 × 10^−9^) in childhood (Fig. 4A). Conversely, neonatal or infantile acquired causes of epilepsy were more likely in the non-genetic group, including cerebral haemorrhage (HP:0001342, OR 0.02, 95% CI: 0.01–0.06, *P*_adj_ = 1.81 × 10^−23^). Interestingly, we found strong signals for renal insufficiency in neonates and infants (HP:0000083, OR 35.86, 95% CI: 21.97–62.91, *P*_adj_ = 2.08 × 10^−168^) and osteoporosis in adults (HP:0000939, OR Inf, 95% CI: 3.58–Inf, *P*_adj_ = 5.17 × 10^−3^) with known or likely genetic epilepsy. We included four common childhood comorbidities that were not expected to be enriched in cases (hyperglycaemia, parasomnia, otitis media and allergic rhinitis) as controls. Across the age range, none of these features were enriched in cases.

Likewise, we hypothesized that the treatment rationale of genetic epilepsy changes over the age range. Data on 15 003 prescriptions were available for 365/503 (73%) of the study cohort. Individuals with likely genetic epilepsy were more likely to receive long-term drug therapy (UMLS:C2911188, OR 4.32, 95% CI: 2.47–7.70, *P*_adj_ = 1.52 × 10^−7^) and received significantly more prior and concurrent anti-seizure medications (ASM) [mean unique ASMs per person 4.47 (SD = 2.90) versus 3.19 (SD = 2.24), two-sided *t*-test, *P*_adj_ = 3.04 × 10^−6^]. Importantly, they received more prescriptions for rescue medication (benzodiazepines) [mean prescriptions per person 11.90 (SD = 14.70) versus 8.14 (SD = 13.40), two-sided *t*-test, *P*_adj_ = 0.048]. Likewise, prescription patterns for ASM differed between the two groups and changed across age intervals. Individuals with a likely genetic aetiology received first-line ASMs (levetiracetam, valproic acid) earlier and broad-spectrum ASMs (topiramate, lacosamide) later in life. Also, they were more likely to be exposed to syndrome-specific ASMs with potentially severe side effects (vigabatrin, felbamate) (Fig. 4B).

### Data-driven identification of genetic individuals reveals unexpected and underrecognized aetiologies

The cohort definition introduced in the discovery analysis presented above distinguishes case and control participants by stratification with a NLP algorithm but also by whether or not they have received genetic testing. We consider this to be a reasonable definition, given that the clinical characteristics, patterns of healthcare utilization and differences in medical treatment identified above may thus be used to identify individuals that should receive genetic testing. However, we acknowledge two sources of potential bias: firstly, the discovery cohort may contain individuals that appear to be likely genetic, e.g. developmental delay, intractable seizures and a variant of unknown significance in an epilepsy-associated gene, but which should not be strictly considered as ‘genetic cases’ in the absence of a (likely) pathogenic variant according to ACMG classification. We note that this was by design in order to ensure a large and thus well-powered discovery cohort. Secondly, individuals that received genetic testing are more likely to share certain characteristics simply by virtue of fulfilling the diagnostic criteria needed to prompt testing in the first place. We therefore conducted manual chart review on all 259 individuals previously stratified as ‘likely genetic’ by our NLP algorithm. Overall, 205/259 (79.15%) individuals were found to have (likely) pathogenic variants or variant of unknown significance (VUS) in an epilepsy-associated gene. Forty of 259 (15.44%) were found to be false-positive, e.g. negative whole-exome sequencing or genetic testing for a disorder unrelated to epilepsy, while the remaining 14/259 (5.41%) did not receive sufficient genetic testing (e.g. only single-gene testing or microarray analysis).

We proceeded to validate our key findings by manual chart review, first focusing on individuals with the potentially novel clinical associations outlined above: renal insufficiency in neonates and infants, osteoporosis in adulthood and genitourinary abnormalities (Table 2). Interestingly, neonatal and infantile renal insufficiency or genitourinary abnormalities were primarily observed in rare congenital multisystem disorders (e.g. Kabuki syndrome, Warburg Micro syndrome, DiGeorge syndrome or Cornelia de Lange syndrome) and microdeletion or duplication syndromes (e.g. chromosome 15q11-q13 duplication, Prader–Willi syndrome). In all cases, osteoporosis was confirmed by a DEXA scan and was found in childhood hypophosphatasia, combined oxidative phosphorylation deficiency and Dravet syndrome. Canonical genetic epilepsy syndromes (e.g. tuberous sclerosis complex 1, *CDKL5*-related developmental and epileptic encephalopathy 2 or ion channel disorders) comprised only the minority of cases (Table 2). The full list of individuals with confirmed genetic epilepsy syndromes is available as Supplementary Table 3.

**Table 2:** Results of manual chart view to confirm key novel findings

Clinical association	Syndrome
Osteoporosis (HP:0000939)	Combined oxidative phosphorylation deficiency, type 15 (MIM #614947)
	Hypophosphatasia, childhood (MIM #241510)
	Dravet syndrome (MIM #607208)
Renal insufficiency (HP:0000083)	Down syndrome (MIM #190685)
	Developmental and epileptic encephalopathy 2 (*CDKL5*, MIM #300672)
	Tuberous sclerosis 1 (MIM #191100)
	Warburg micro syndrome 1 (*RAB3GAP2*, MIM #600118)
	Kabuki syndrome (*KMT2D*, MIM #147920)
	Pontocerebellar hypoplasia (*TSEN54*, MIM #608755) and Alport syndrome 2 (*COL4A3*, MIM #203780)
	2q and 15q deletion (not specified), VACTERL association (MIM #192350)
	Developmental and epileptic encephalopathy 18 (*SZT2*, MIM #615476)
	Microdeletion syndrome (20p 12.2–12.3, 1q21.1–21.1)
	*COL4A1*-related schizencephaly (MIM #120130)
	Schimmelpenning–Feuerstein–Mims syndrome (*KRAS*, MIM #163200)
	DiGeorge syndrome (*TBX1*, MIM #188400)
Hypospadias (HP:0000047)	Cornelia de Lange syndrome (*NIBPL*, MIM #122470)
	Kabuki syndrome (*KMT2D*, MIM #147920)
	Chromosome 15q11-q13 duplication syndrome (MIM #608636)
	Septo-optic dysplasia syndrome (*HESX1*, MIM #182230)
Cryptorchidism (HP:0000028)	Down syndrome (MIM #190685)
	Cornelia de Lange syndrome (*NIBPL*, MIM #122470)
	Prader–Willi syndrome (15q11-q13del, MIM #176270)
	Kabuki syndrome
	Chromosome 15q11-q13 duplication syndrome (MIM #608636)
	Tatton-Brown–Rahman syndrome (*DNMT3A*, MIM #615879)
	Neurofibromatosis type 1 (*NF1*, MIM #162200)
	Developmental and epileptic encephalopathy 1 (ARX, MIM #308350)
	Septo-optic dysplasia syndrome (*HESX1*, MIM #182230)

MIM, Mendelian Inheritance in Man.

### Validation in controls who received negative genetic testing confirms key findings

Some of the same characteristics that inform the decision to order genetic testing (discovery cohort) may also help to differentiate individuals who received a genetic diagnosis from those who remained undiagnosed (validation cohort). If there were salient clinical characteristics of genetic epilepsy, we would expect to observe them across both cohorts. Based on manual chart review, we excluded 35 individuals with variants of unknown significance, resulting in a strict subset of 170 cases with a confirmed genetic diagnosis who were matched to an independent control cohort of 174 individuals who have received negative genetic testing. We observed the same relationships in age-dependent healthcare utilization: individuals with confirmed genetic epilepsies were younger at all encounters with the healthcare system [mean age 5.23 (SD = 4.92) years versus 5.96 (SD = 5.17), two-sided *t*-test, *P* < 0.0001]. They were also younger when they were first diagnosed with epilepsy [age at ICD-10 G40.-, mean age 1.77 (SD = 1.41) years versus 2.26 (SD = 1.77), two-sided *t*-test, *P* < 0.0001] (Supplementary Fig. 3). Most notably, we observed the same increase in healthcare utilization during transition from paediatric to adult care [annual encounters during transition 6.74 (SD = 2.96) versus 3.90 (SD = 2.53), two-sided *t*-test, *P* = 1.84 × 10^−5^]. These were primarily driven by severe somatic comorbidities (autism and congenital malformations; Supplementary Fig. 3).

Thus, individuals with genetic epilepsy had more frequent encounters with the healthcare system. We further analysed total healthcare-associated costs from an internal claims database to independently confirm this increased healthcare resource utilization and to test for financial burden. Individuals with genetic epilepsy had higher costs for each outpatient encounter (median cost before insurance adjustment 390 USD, IQR = 155 USD, versus 370 USD, IQR = 179 USD; two-sample Wilcoxon test, *P* = 0.0215) and higher annual healthcare-related costs than those with non-genetic epilepsy (median annual cost before insurance adjustment 13 371 USD, IQR = 39 186 USD, versus 9898 USD, IQR = 29 161 USD; two-sample Wilcoxon test, *P* = 0.0178). Likewise, costs in the genetic group increased around the age of transition (Supplementary Fig. 4).

**Figure 5:**
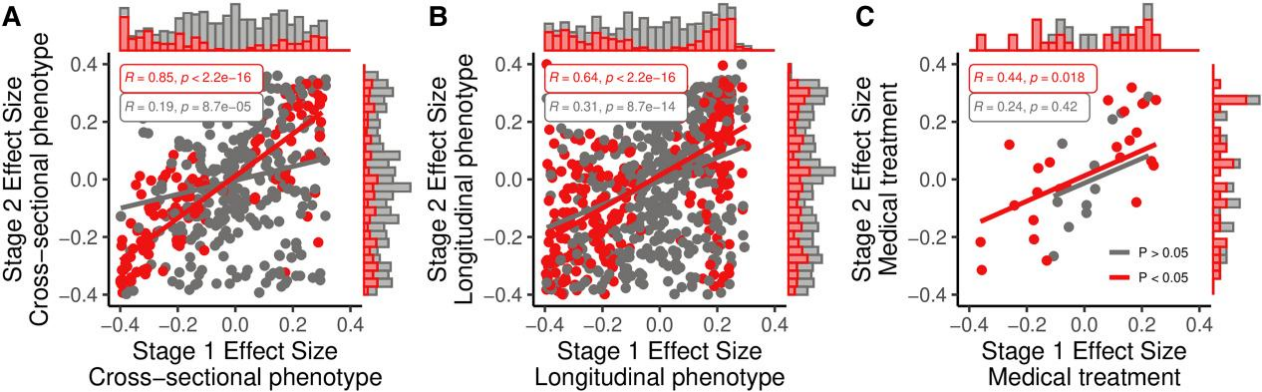
**Original effect size estimates versus replication effect sizes.** (**A**) Cross-sectional phenotype associations. (**B**) Longitudinal phenotype associations. (**C**) Medical treatment associations. Each dot corresponds to a single hypothesis and is coloured red if the null hypothesis was rejected at a nominal significance level (*P* < 0.05). Results for tests of correlation between paired samples are shown using Pearson’s product moment correlation coefficient.

On cross-sectional analysis of clinical characteristics in the validation cohort, we confirmed novel associations such as abnormalities of the genitourinary system (HP:0000119; OR 2.01, 95% CI: 1.78–2.28, *P*_adj_ = 3.27 × 10^−27^) and known associations such as hyperactivity (HP:0000752, OR 2.01, 95% CI: 1.64–4.31, *P*_adj_ = 0.016) or attention deficit (HP:0007018; OR 2.62, 95% CI: 1.64–4.30, *P*_adj_ = 0.017; Supplementary Fig. 5). Interestingly, we found no associations with simple febrile seizures (HP:0011171; depleted in the discovery cohort) but an enrichment for complex febrile seizures (HP:0011172; OR 6.94, 95% CI: 2.45–27.05, *P*_adj_ = 0.011). Meanwhile, causes of symptomatic epilepsy such as cerebral haemorrhage were depleted (HP:0001342, OR 0.02, 95% CI: 0.01–0.09, *P*_adj_ = 1.67 × 10^−14^). Two UMLS concepts were significantly different between groups: ‘Other specified chromosome abnormalities’ (UMLS:C0478107, OR 9.11, 95% CI: 2.90–38.20, *P*_adj_ = 6.60 × 10^−6^) and ‘Other generalized epilepsy and epileptic syndromes, intractable’ (UMLS:C2875116, OR 6.24, 95% CI: 2.69–15.6, *P*_adj_ = 1.52 × 10^−6^). We conducted statistical analysis of the reproducibility of all tests for cross-sectional clinical characteristics (i.e. association with one of 1216 HPO terms; Fig. 5A). One hundred sixty of 282 (56.73%) of nominally significant associations (*P* < 0.05) were reproduced with the original effect direction. Effect size estimates as odds ratios were converted to point-biserial correlation *r*, which are bounded and thus more readily interpretable.^26^ Mean effect size estimates were *r* = −0.0756 (SD 0.271, median −0.0423) for cases and *r* = −0.0694 (SD 0.282, median −0.0487) for controls (two-sided *t*-test, *P* = 0.666). The effect size estimates of 361/776 (46.52%) clinical associations in the discovery cohort were within the 95% CI of the effect size estimates in the validation cohort. Overall, effect size estimates for nominally significant tests were strongly correlated (Pearson’s *R* = 0.85, *P* < 2.2 × 10^−16^; Fig. 5A).

We further replicated key findings on the longitudinal clinical characteristics of individuals with confirmed genetic epilepsy across the age groups defined above. We found the same relative enrichment for recurrent infections, constipation and dehydration in childhood and depletion for cerebral haemorrhage and meningitis in the neonatal period and infancy. This included the novel signals for renal insufficiency (HP:0000083, OR 83.76, 95% CI: 45.33–175.48, *P*_adj_ = 1.51 × 10^−241^) and osteoporosis (HP:0000939, OR Inf, 95% CI: 9.76–Inf, *P*_adj_ = 1.69 × 10^−9^; Supplementary Fig. 6). Overall, 452/1021 (44.27%) of nominally significant associations (*P* < 0.05), each representing a single HPO term within a given time interval, were reproduced with the original effect direction. Mean effect size estimates were *r* = −0.120 (SD = 0.265, median −0.102) for cases and *r* = −0.0446 (SD = 0.275, median −0.0360) for controls (two-sided *t*-test, *P* = 1.18 × 10^−14^). The effect size estimates of 570/2322 (24.55%) clinical associations in the discovery cohort (stage 1) were within the 95% CI of the effect size estimates in the validation cohort (stage 2). Effect size estimates for nominally significant tests were strongly correlated (Pearson’s *R* = 0.64, *P* < 2.2 × 10^−16^; Fig. 5B).

Lastly, we observed the same general medical treatment patterns in individuals with confirmed genetic epilepsy. Data on 12 893 prescriptions were available across 266/344 (77%) of the study cohort. Individuals with confirmed genetic epilepsy received significantly more prior and concurrent ASM [mean unique ASMs per person 4.88 (SD = 2.92) versus 3.21 (SD = 2.26), two-sided *t*-test, *P*_adj_ = 4.51 × 10^−7^]. They also received more prescriptions for rescue medication [mean prescriptions per person 13.30 (SD = 15.40) versus 9.09 (SD = 10.80), two-sided *t*-test, *P*_adj_ = 0.038]. Individuals with genetic epilepsy received first-line ASMs (levetiracetam, valproic acid) early and were more likely to be exposed to syndrome-specific ASMs (vigabatrin, cannabidiol, rufinamide) (Supplementary Fig. 6). The UMLS concept annotation for long-term drug therapy was not significantly different between groups. Twenty-nine of 51 (56.86%) of nominally significant associations (*P* < 0.05), each representing an ASM prescription within a given time interval, were reproduced with the original effect direction. Mean effect size estimates were *r* = −0.0840 (SD = 0.286, median −0.0541) for cases and *r* = −0.0449 (SD = 0.226, median −0.0615) for controls (two-sided *t*-test, *P* = 0.429). The effect size estimates of 23/77 (29.87%) clinical associations in the discovery cohort (stage 1) were within the 95% CI of the effect size estimates in the validation cohort (stage 2). The effect size estimates for nominally significant tests were correlated (Pearson’s *R* = 0.44, *P* = 0.018; Fig. 5C).

## Discussion

Healthcare resource utilization and disease burden in individuals with genetic epilepsy are not well-understood, as these disorders are individually rare. This knowledge gap is particularly evident for non-neurological phenotypes given current referral paths, where care centres around paediatric neurology. Previous studies have focussed on single-gene and gene family cohorts^13,14^ or attempted to address this problem by observing direct costs or quality of life from insurance claims and online surveys.^27^ Here, we instead utilized NLP and deep computational phenotyping across a large healthcare system to identify a longitudinal cohort of individuals with childhood-onset genetic epilepsy and matched controls with non-genetic epilepsy. This approach was particularly successful in identifying system-level factors beyond the core neurological phenotype: we found several markers of increased healthcare resource utilization. Individuals with genetic epilepsy were more likely to be admitted to inpatient services or the emergency department. They had more frequent encounters with the healthcare system, more diagnoses per encounter and higher costs per encounter. Importantly, they were seen significantly more often during the transition from paediatric to adult care, likely because of more severe comorbidities, and had an increase in healthcare-related costs during this time. Transition is a critical period that requires multidisciplinary care teams.^25^ This study provides objective evidence to support the need for transition care, which was previously limited.^25^

Finding clinical features associated with genetic epilepsy improves patient selection and cost-effectiveness for genetic testing by increasing the pre-test probability of a positive finding.^9^ Previous studies have demonstrated how deep longitudinal data from healthcare systems can be leveraged to characterize monogenic syndromes.^13-15^ Here, we validated previous findings, including independent statistical support for several known predictors: intractable seizures, behavioural abnormalities, autism, developmental delay, intellectual disability, abnormalities of movement (including ataxia), pharmacoresistance (long-term drug therapy or exposure to multiple ASMs) and others. Conversely, we found individuals with probable causes of acquired epilepsy (e.g. cerebral haemorrhage) less likely to have a genetic diagnosis. These factors have been described in studies of clinical sequencing yield, which are reflected in current practice guidelines that recommend genetic testing, preferably whole-exome sequencing, in any individual with seizures and intellectual disability.^9,10^

Our data-driven whole-phenome approach identified individuals with disorders that commonly present with seizures but which are not traditionally considered epilepsy syndromes. These include rare congenital multisystem disorders and chromosomal disorders, which have received less attention when compared with the aetiology-specific developmental and epileptic encephalopathies caused by ion channel or transporter disorders.^28^ In our study, these individuals contributed towards a novel signal for genitourinary abnormalities including congenital malformations. This clinical aspect can therefore be kept in mind for children with dysmorphic and chromosomal disorders. Further, longitudinal phenotyping revealed markers of disease burden and age-specific general clinical features, e.g. a higher likelihood of feeding difficulties, dehydration, constipation, recurrent infections or hypoxaemia in childhood. These are clinical issues commonly seen in neurodevelopmental disorders.^29^ Likewise, data from medical prescriptions demonstrated group-level differences in disease burden and severity. Individuals with genetic epilepsy were more likely to receive long-term drug therapy or multiple ASMs, with more prescriptions for rescue medication and earlier exposure to broad-spectrum or syndrome-specific ASMs, in line with previous evidence of polypharmacy in this vulnerable group.^30^

This study leveraged >180 000 concept annotations across >4000 person-years, utilizing deep computational phenotyping and well-matched controls to provide statistical power for our analysis. The study site, an integrated Level 4 Adult and Paediatric Epilepsy Centre, enabled us to achieve longer follow-up than previous studies, spanning the critical transition period. The cohort definition was based on gold standard criteria, with orthogonal validation by another scoring system and manual chart review. Our hypothesis-free ontological reasoning approach was designed to minimize the effect of bias or unaccounted confounders. However, this study only reports on statistical associations and cannot be used to establish causality between genetic disorders and their comorbidities. We note that some of the associations, e.g. osteoporosis and renal insufficiency, may be secondary due to malnutrition, drug side effects or multiorgan dysfunction. Likewise, the increased healthcare utilization of individuals with genetic epilepsy may be partially related to environmental factors or geographic access to our healthcare system. A potential risk of misclassification bias may be addressed by extending recent work on machine learning–based patient identification.^31^ While this study was conducted in a large multicentre healthcare system, we were still limited to a US population sample. As demonstrated above, independent replication of findings and external validity across different healthcare systems remains a central challenge, mainly due to different patient populations. Indeed, our study setting may be somewhat more likely to attract complex or refractory cases that benefit from specialized care. Thus, our study cohort is likely skewed towards more severe cases. Lastly, healthcare systems as real-world data sources will always be subject to key limitations, including variable documentation quality, billing or procedural practice changes and discontinuous healthcare usage.^32^ Prospectively, case identification from real-world data may be improved by more closely aligning the ICD structure with the ILAE classifications of seizures and epilepsy syndromes.

Future research directions may include deep computational phenotyping in clinical sequencing yield studies to power gene discovery and confirm the clinical utility of the identified statistical associations. Finally, an improved understanding of the longitudinal disease trajectories of these individuals will contribute towards both a timely diagnosis and syndrome-specific disease forecasting models.^33^

## Supplementary Material

fcae090_Supplementary_Data

## Data Availability

Deidentified individual participant data can be made available upon reasonable requests to the corresponding author. The prerequisite for data sharing is a data transfer agreement approved by the legal departments and institutional review board of the requesting researcher. After proposal approval, data can be shared through a secure online platform. All code used for data analysis and visualization is available at https://github.com/christianbosselmann/UMLS-HPO.
