# Supplementary material for: Healthcare utilization and clinical characteristics of genetic epilepsy in electronic health records
Source: Brain Commun. 2024 Mar 14;6(2):fcae090. doi: 10.1093/braincomms/fcae090 (PMC10959483; doi:10.1093/braincomms/fcae090)
Supplement: fcae090_Supplementary_Data [file fcae090_supplementary_data.pdf]

Supplementary Material for "Healthcare utilization and clinical characteristics of genetic epilepsy in electronic health records"

Christian M Boßelmann[1,2], Alina Ivaniuk[1,2], Mark St John[1,2], Sara C Taylor[3], Gokul Krishnaswamy[3], Alex Milinovich[4], Costin Leu[1,5,8,9], Ajay Gupta[2], Elia M Pestana-Knight[2], Imad Najm[2], Dennis Lal[1,2,6-9*]

[1]Genomic Medicine Institute, Lerner Research Institute, Cleveland Clinic, Cleveland, OH, USA. [2]Epilepsy Center, Neurological Institute, Cleveland Clinic, Cleveland, OH, USA. [3]Neurological Institute, Cleveland Clinic, Cleveland, OH, USA. [4]Department of Quantitative Health Sciences, Cleveland Clinic, Cleveland, OH, USA. [5]Department of Clinical and Experimental Epilepsy, Institute of Neurology, University College London, London, UK. [6]Stanley Center for Psychiatric Research, Broad Institute of Harvard and M.I.T., Cambridge, MA, USA. [7]Cologne Center for Genomics (CCG), University of Cologne, Cologne, DE. [8]Department of Neurology, The University of Texas Health Science Center at Houston, Houston, TX, USA. [9]Center for Neurogenetics, The University of Texas Health Science Center at Houston, Houston, TX, USA.

SUPPLEMENTARY FIGURE 1: ADDITIONAL VALIDATION WITH THE PHEINDEX CRITERIA..2

SUPPLEMENTARY FIGURE 2: ONTOLOGICAL REPRESENTATION AND PROPAGATION 3

SUPPLEMENTARY TABLE 1: NATURAL LANGUAGE PROCESSING ALGORITHM 4

SUPPLEMENTARY TABLE 2: CLINICAL CHARACTERISTICS OF THE DISCOVERY COHORT ..5

SUPPLEMENTARY TABLE 3: RESULTS OF MANUAL CHART REVIEW 7

SUPPLEMENTARY FIGURE 3: VALIDITY ANALYSIS OF AGE-DEPENDENT HEALTHCARE UTILIZATION...17

SUPPLEMENTARY FIGURE 4: ANALYSIS OF HEALTHCARE-RELATED COSTS...........................18

SUPPLEMENTARY FIGURE 5: VALIDITY ANALYSIS OF KNOWN AND NOVEL CLINICAL CHARACTERISTICS...19

SUPPLEMENTARY FIGURE 6: VALIDITY ANALYSIS OF LONGITUDINAL CLINICAL CHARACTERISTICS AND TREATMENT PATTERNS 20

Supplementary Figure 1: Additional validation with the PheIndex criteria

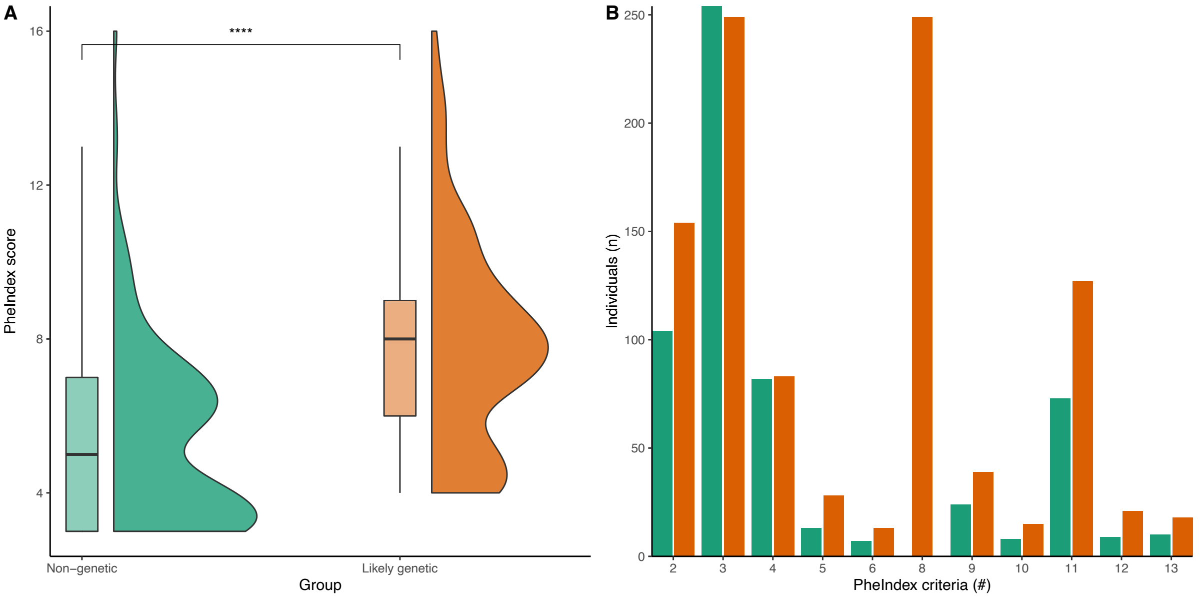

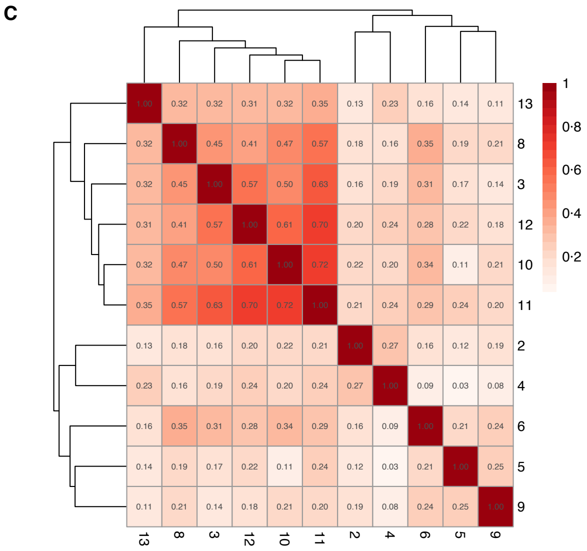

D

Criterion	Description
1	Prolonged stay in the NICU
2	Prolonged or multiple hospitalizations
3	Visits or consults with multiple specialists
4	Multiple emergency room visits
5	Feeding support
6	Respiratory support
7	Imaging
8	Genetic diagnostic tests
9	Metabolic diagnostic tests
10	In−hospital death
11	Developmental delay
12	Diagnosis corresponding to metabolic diseases
13	Heart surgeries

Supplementary Figure 1. PheIndex scores for individuals with likely genetic epilepsy and individuals with non-genetic epilepsy. A: Likely genetic patients had a significantly higher average PheIndex score (7·84 SD 2.71 vs. 5.41 SD 2.41, two-sample t-test, $p = 9.79 \times 10^{-24}$). **B:** Distribution of PheIndex criteria for both groups. Data for criterion 1 (prolonged stay in the neonatal intensive care unit, NICU) and criterion 7 (imaging) were not available. Note that criterion 3 is true for both groups (due to the study setting, a multidisciplinary epilepsy centre), and criterion 8 is true for cases only (due to the eligibility criteria including genetic testing). **C:** Heatmap of co-occurrence of PheIndex criteria in the study cohort. Developmental delay, in-hospital death, metabolic disease, consults with multiple specialists, and genetic diagnostic tests formed a single cluster. Labels show the pairwise frequency at which two criteria occur in the same individual. Hierarchical clustering on cosine similarity between sets of PheIndex criteria. **D:** List of PheIndex criteria.

Supplementary Figure 2: Ontological representation and propagation

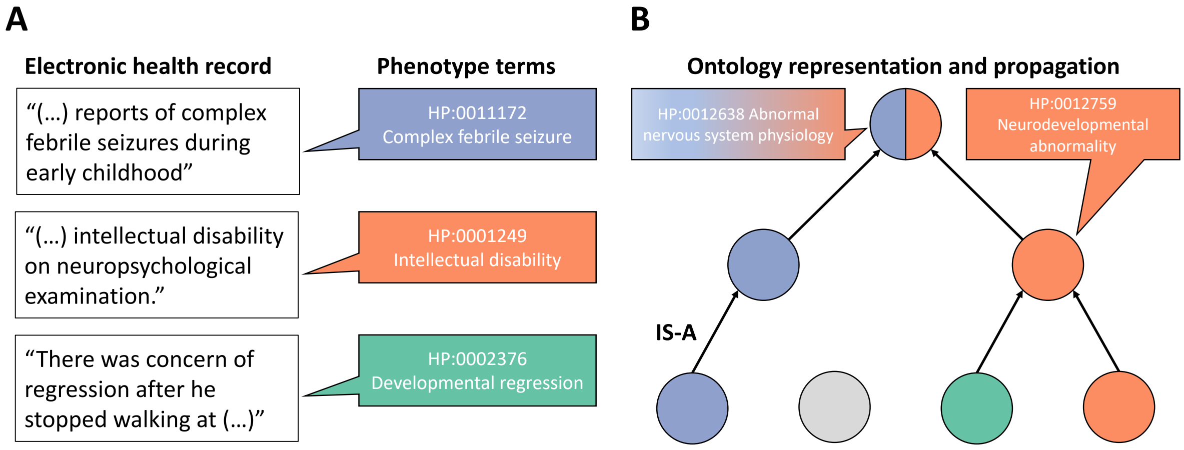

Supplementary Figure 2. A: Clinical descriptions from provider notes, billing codes, and other text sources are extracted from the electronic health record (EHR) and mapped to standardized human phenotype ontology (HPO) terms. Each term represents a node in a directed acyclic graph, where edges between nodes are IS-A (parent-child) relationships. Terms are propagated along these edges, where each parent term is included in the set of terms until the root node (i.e., HP:0000001 All) for each original term is included in the set. **B:** Intuitively, this allows the data to reflect the hierarchical ordering of clinical findings, i.e., that terms like developmental regression and intellectual disability both fall under the broader category of neurodevelopmental abnormality, which in turn is an abnormality of nervous system physiology. Figure adapted from Boßelmann et al.[1]

Supplementary Table 1: Natural language processing algorithm

Participants eligible for stratification were filtered for electronic health record (EHR) notes containing a set of prespecified epilepsy-related genes: *CACNA1A, CACNA1E, CDKL5, DEPDC5, GABRA1, GABRB2, GABRB3, GABRG2, GNAO1, GRIN1, GRIN2A, GRIN2B, GRIN2D, KCNA1, KCNA2, KCNB1, KCNQ2, KCNT1, MECP2, MTOR, NPRL2, NPRL3, PCDH19, PIK3CA, SCN1A, SCN2A, SCN3A, SCN8A, SLC13A5, SLC2A1, SLC6A1, STX1B, STXBP1, SYNGAP1, TSC1, TSC2*. These notes (n = 230) were then manually reviewed for sets of phrases that either confirmed or disproved that the individual had a pathogenic or likely pathogenic variant or variant of unknown significance (VUS) (Supplementary Table 1). For each phrase, we assigned a score of negatively correlated (-1) or positively correlated (+1) with the presence of a genetic aetiology (Supplementary Table 1). For each individual, phrases across all EHR notes were aggregated to calculate the final sum score. Note sections pertaining to family history or insurance approval requests were removed from analysis. Any individual with a sum score > 0 was considered to have a likely genetic aetiology.

Negatively correlated phrases	Positively correlated phrases
'sequencing {gene} negative'	'confirmed {gene} mutation'
'{gene} negative mutation'	'positive {gene}.* c.'
'{gene} mutation negative'	'{gene} gene mutation'
'sequencing {gene} normal'	'{gene} genetic mutation'
'genetic test.*{gene} normal'	'mutation {gene} gene'
'{gene} negative'	'mutation gene {gene}'
'{gene} negative'	'mutation {gene} gene'
'negative {gene}'	'{gene} gene sequencing identified mutation'
'negative mutation analysis of {gene}'	'{gene} gene pathogenic mutation'
'{gene} test negative'	'pathogenic {gene}.{0,10}mutation'
'gene test {gene} neg '	'variants unknown significance {gene}'
'negative mutation {gene}'	'pathogenic variant {gene}'
'{gene} gene mutation negative'	'variants uncertain significance detected {gene}'
'{gene} gene test negative'	'remarkable {gene}'
'No mutations {0,10} {gene}'	'{gene} mutation .{0,10} [0-9][ACG][<>]'
'tested genetic.{0,10}{gene} neg '	'{gene} Variant Transition Nucleotide'
	'CAG repeats {gene}'
	'heterozygous missense.{0,10}{gene}'
	'heterozygous.*variant.{0,10}{gene}'
	'sequence alteration .{0,10}{gene}'
	'Gene panel show.{0,10}{gene} variant'
	'Gene panel positive {gene}'
	'{gene}.[c]\.[0-9] .* VUS'
	'Heterozygous VUS {gene}'
	'variant {gene} gene identified'
	'genetic mutation detected.*heterozygous.{0,10} {gene}'
	'partial deletion {gene} gene, paternally inherited'
	'Partial deletion {gene} gene, maternal inherited'
	'Genetic test showed VUS {gene}'

Supplementary Table 1. Phrases used in the natural language processing algorithm to identify individuals with a likely genetic etiology. Abbreviations: VUS – variant of unknown significance.

Supplementary Table 2: Clinical characteristics of the discovery cohort

ICD-10	Likely genetic	Not genetic	Description
G40.419	51	9	Other generalized epilepsy and epileptic syndromes, intractable, without status epilepticus
G40.319	37	20	Generalized idiopathic epilepsy and epileptic syndromes, intractable, without status epilepticus
G40.824	31	12	Epileptic spasms, intractable, without status epilepticus
G40.119	30	29	Localization-related (focal) (partial) symptomatic epilepsy and epileptic syndromes without status epilepticus
G40.309	30	22	Generalized idiopathic epilepsy and epileptic syndromes, not intractable, without status epilepticus
G40.802	24	22	Other epilepsy, not intractable, without status epilepticus
G40.909	21	16	Epilepsy, unspecified, not intractable, without status epilepticus
G40.911	21	3	Epilepsy, unspecified, intractable, with status epilepticus
G40.109	20	17	Localization-related (focal) (partial) symptomatic epilepsy and epileptic syndromes without status epilepticus
R56.9	19	16	Unspecified convulsions
G40.209	18	10	Localization-related (focal) (partial) symptomatic epilepsy and epileptic syndromes with complex partial seizures, not intractable, without status epilepticus
G40.901	18	10	Epilepsy, unspecified, not intractable, with status epilepticus
R56.00	17	17	Simple febrile convulsions
G40.822	15	6	Epileptic spasms, not intractable, without status epilepticus
G40.814	14	1	Lennox-Gastaut syndrome, intractable, without status epilepticus
G40.211	11	3	Localization-related (focal) (partial) symptomatic epilepsy and epileptic syndromes with complex partial seizures, intractable, with status epilepticus
G40.409	11	3	Other generalized epilepsy and epileptic syndromes, not intractable, without status epilepticus
F84	10	7	Pervasive developmental disorders
G40.919	9	11	Epilepsy, unspecified, intractable, without status epilepticus
R56.01	9	9	Complex febrile convulsions
G40.301	8	3	Generalized idiopathic epilepsy and epileptic syndromes, not intractable, with status epilepticus
G40.401	8	1	Other generalized epilepsy and epileptic syndromes, not intractable, with status epilepticus
G40.42	8	0	Cyclin-Dependent Kinase-Like 5 Deficiency Disorder
G40.834	8	0	Dravet syndrome, intractable, without status epilepticus
G40	7	6	Epilepsy and recurrent seizures
G40.A19	7	2	Absence epileptic syndrome, intractable, without status epilepticus
G40.83	7	0	Dravet syndrome
F84.0	6	6	Autistic disorder
G40.201	6	6	Localization-related (focal) (partial) symptomatic epilepsy and epileptic syndromes with complex partial seizures, not intractable, with status epilepticus
G40.219	6	6	Localization-related (focal) (partial) symptomatic epilepsy and epileptic syndromes with complex partial seizures, intractable, without status epilepticus
R56.1	6	4	Post traumatic seizures
G40.509	6	3	Epileptic seizures related to external causes, not intractable, without status epilepticus
G40.801	6	3	Other epilepsy, not intractable, with status epilepticus
G40.311	6	1	Generalized idiopathic epilepsy and epileptic syndromes, intractable, with status epilepticus
G40.89	5	1	Other seizures
G40.009	4	11	Localization-related (focal) (partial) idiopathic epilepsy and epileptic syndromes with seizures of localized onset, not intractable, without status epilepticus
G40.A09	4	9	Absence epileptic syndrome, not intractable, without status epilepticus
G40.001	4	3	Localization-related (focal) (partial) idiopathic epilepsy and epileptic syndromes with seizures of localized onset, not intractable, with status epilepticus
F84.8	4	2	Other pervasive developmental disorders
G40.011	4	2	Localization-related (focal) (partial) idiopathic epilepsy and epileptic syndromes with seizures of localized onset, intractable, with status epilepticus
G40.821	4	2	Epileptic spasms, not intractable, with status epilepticus
G40.823	4	1	Epileptic spasms, intractable, with status epilepticus
F84.2	4	0	Rett's syndrome
G40.803	3	4	Other epilepsy, intractable, with status epilepticus

G40.411	3	0	Other generalized epilepsy and epileptic syndromes, intractable, with status epilepticus
G40.501	2	2	Epileptic seizures related to external causes, not intractable, with status epilepticus
G40.812	2	2	Lennox-Gastaut syndrome, not intractable, without status epilepticus
G40.833	2	0	Dravet syndrome, intractable, with status epilepticus
G40.B09	2	0	Juvenile myoclonic epilepsy, not intractable, without status epilepticus
G40.111	1	2	Localization-related (focal) (partial) symptomatic epilepsy and epileptic syndromes with simple partial seizures, intractable, with status epilepticus
G40.019	1	1	Localization-related (focal) (partial) idiopathic epilepsy and epileptic syndromes with seizures of localized onset, intractable, without status epilepticus
G40.B19	1	1	Juvenile myoclonic epilepsy, intractable, without status epilepticus
G40.10	1	0	Localization-related (focal) (partial) symptomatic epilepsy and epileptic syndromes with simple partial seizures, not intractable
G40.31	1	0	Generalized idiopathic epilepsy and epileptic syndromes, intractable
G40.811	1	0	Lennox-Gastaut syndrome, not intractable, with status epilepticus
G40.A01	1	0	Absence epileptic syndrome, not intractable, with status epilepticus
F84.5	0	1	Asperger's syndrome

Supplementary Table 2. Diagnoses of the discovery cohort. The epilepsy diagnoses associated with the participants from the matched case-control discovery cohort (n = 503) are shown as ICD-10 codes for the case ("Likely genetic") and control ("Not genetic") groups, respectively, sorted by overall prevalence. Codes are shown if they are grouped under G40 ("Epilepsy and recurrent seizures"), R56 ("Convulsions, not elsewhere classified"), or F84 ("Pervasive developmental disorders). Note that individuals may be assigned more than one ICD-10 code over the duration in the healthcare system.

Supplementary Table 3: Results of manual chart review

ID	*Age (y)*	*Sex*	*Genetic aetiology*	Gene or *chromosomal abnormality*	*Phenotype*	Comment
1	18	M	No	NA	ADHD, PDD	Microarray negative, WES pending
2	13	M	No	NA	GE, ASD	Suspected sucrase isomaltase deficiency
3	13	M	Yes	*SCN1A*	Dravet syndrome	
4	13	F	Yes	*MECP2*	Rett syndrome, atypical, MIM 312750	
5	12	M	Yes	*CTNNB1*	Neurodevelopmental disorder with spastic diplegia and visual defects, MIM 615075	
6	12	M	No	NA	FE, ADD, DEE	Microarray and FraX negative
7	11	M	Yes	*MTCO1*	NA	
8	11	M	No	*DEE*	NA	Microarray negative, WES pending
9	11	M	Yes	*CDKL5*	CDD, LGS	
10	11	M	Yes	*POLG*	DEE	
11	10	M	Yes	*NIPBL*	Cornelia de Lange syndrome	
12	14	M	Yes	*TCF4*	Pitt-Hopkins syndrome	
13	10	M	Yes	*KCNT1*	DEE14	
14	18	F	No	NA	DEE, cortical vision loss, microcephaly	Microarray and single-gene testing negative
15	6	F	Yes	del4q28.3	DEE	
16	17	F	No	NA	NA	Tested for family history of *PRPF31* (retinitis pigmentosa)
17	18	F	No	NA	ADHD, cerebellar atrophy and ataxia	Microarray negative, WES pending
18	16	F	Yes	*NSD1*	Sotos syndrome	
19	17	M	Yes	46 XY / 47 XYY mosaicism, dupXp28, del4q35.2, del6p25.1	DEE, ataxia	
20	16	F	Yes	*ALDH7A1*	PDE	
21	17	F	Yes	*CSNK1E*	Myoclonic epilepsy	
22	15	M	Yes	NA	DEE	Chromosomal abnormality, not specified; VUS *NSDHL*
23	15	M	Yes	*CLN1*	Neuronal ceroid lipofuscinosis	

24	14	M	Yes	*TSC2*	TSC	
25	14	M	Yes	*TSC2*	TSC	
26	14	F	Yes	transXp;2p	DEE	Complex chromosomal abnormality
27	13	F	Yes	*TSC2*	TSC	
28	14	M	No	NA	HME	Microarray and single-gene testing (TSC1/2) negative; likely somatic mosaicism
29	14	M	Yes	*MTFMT*	Leigh syndrome (COXPD15)	
30	13	F	Yes	*CACNA1A*	Developmental and epileptic encephalopathy 42, MIM 617106	
31	13	M	Yes	*ACADS*	SCAD, MIM 201470	
32	12	F	Yes	*TSC2*	TSC	
33	14	F	Yes	*SOX2*	Microphthalmia, syndromic 3, MIM 206900	
34	11	M	Yes	*SPATA5*	NEDHSB	
35	13	F	Yes	*KCNQ2*	DEE	
36	12	M	No	NA	FE	Genetic testing for factor V deficiency
37	15	F	Yes	*CDKL5*	CDD	
38	11	M	No	NA	HBB (Hemoglobin C disease)	VUS *FANCD2*
39	11	M	Yes	del9q33, 3p partial monosomy	DEE	
40	12	M	Yes	del10, not specified	ASD, DD	
41	10	F	Yes	*FGFR2*	Beare-Stevenson cutis gyrata syndrome	
42	13	M	Yes	*DMD, FAAH2, OGT, ARGHEF9*	ADHD, DEE	
43	14	F	Yes	*CHRNA4*	ADNLFE	
44	11	M	Yes	dup15q13.3	ASD, DEE	
45	10	M	Yes	*GRIA3*	Intellectual developmental disorder, X-linked syndromic, Wu type, MIM 300699	
46	11	M	Yes	15q11-q13	Prader-Willi syndrome	
47	12	F	No	*RAP3GAP2*	DEE, lissencephaly, spastic quadriparesis	VUS *RAP3GAP2*
48	11	M	No	NA	DEE	WES negative
49	10	M	Yes	*WWOX*	LGS, DEE28	VUS *DOCK7*
50	11	F	Yes	*NUBPL*	Mitochondrial complex I deficiency, nuclear type 21, MIM 618242	

51	10	F	Yes	*SCN1A*	Dravet syndrome	
52	10	F	Yes	15q11.2	Angelman syndrome	
53	3	M	Yes	*GLB1*	GM1-gangliosidosis, type I, MIM 230500	
54	20	F	Yes	*FOXG1*	Rett syndrome, congenital variant, MIM 613454	
55	19	M	Yes	*WDR26*	Skraban-Deardorff syndrome, MIM 617616	
56	26	F	Yes	*TSC2*	TSC	
57	26	F	No	NA	Fibromyalgia	
58	23	M	Yes	*TSC2*	TSC	
59	23	M	Yes	*NR2F1*	Bosch-Boonstra-Schaaf optic atrophy syndrome, MIM 615722	
60	25	M	No	NA	DEE	Microarray negative, WES pending
61	23	F	Yes	*TSC1*	TSC	
62	19	M	Yes	*PPP2R5D*	Intellectual developmental disorder, autosomal dominant 35, MIM 616355	
63	23	F	Yes	*SCN1A*	Dravet syndrome	
64	22	M	Yes	*CACNA1A*	Developmental and epileptic encephalopathy 42, MIM 617106	
65	21	M	Yes	*TNSALP*	Hypophosphatasia, childhood, MIM 241510	
66	22	F	Yes	*ACADs*, not specified	GE, ASD, SCZ	
67	21	M	Yes	*TSC2*	TSC	
68	21	F	Yes	*SCN4A*	Myopathy, not specified	Seizures likely incidental
69	21	M	Yes	*KMT2D*	Kabuki syndrome 1, MIM 147920	
70	22	M	No	NA	GAD, ASD, ID	Suspected mitochondrial disorder, further testing pending
71	21	F	Yes	*TSEN54*	LGS	
72	21	F	Yes	*SCN1A*	Dravet syndrome	
73	21	F	Yes	*TSC*	TSC	
74	20	F	Yes	del6q16.2-q16.3	6q16 microdeletion syndrome	
75	20	F	No	NA	NA	Evaluated for suspected Marfan syndrome
76	11	M	No	NA	ASD, GE	Microarray and targeted panel (Rett) negative, lost to follow-up
77	20	M	No	NA	HSP, FE, ataxia	Panel negative, WES pending

78	22	M	Yes	*SCN1A*	Dravet syndrome	
79	19	F	Yes	*SCN1A*	Dravet syndrome	
80	19	M	No	*DMD*	Duchenne muscular dystrophy	Unclear seizure disorder
81	18	F	No	NA	ISS, DEE	Panel negative, WES pending
82	18	F	No	NA	DEE, hypotonia	Microarray and single-gene testing negative
83	22	F	No	NA	NA	Evaluated for pulmonary hypertension
84	18	F	Yes	*TUBB2A*	Cortical dysplasia, complex, with other brain malformations 5, MIM 615763	
85	15	F	Yes	*SLC2A1*	GLUT1-DS	
86	15	M	Yes	*TSC2*	TSC	
87	15	M	Yes	*NF1*	NF	
88	14	M	Yes	*SCN1A*	Dravet syndrome	
89	22	M	No	NA	DD, hypotonia	Microarray and single-gene testing (*SLC6A8*) negative
90	14	F	Yes	*SCN1A*	Dravet syndrome	
91	13	M	Yes	*GALC*	Krabbe disease, MIM 245200	
92	13	M	Yes	*ALDH7A1*	PDE	
93	12	F	Yes	*KCNQ2*	Seizures, benign neonatal, 1, MIM 121200	
94	12	M	No	NA	GEFS+	Panel negative, WES pending
95	12	M	No	NA	NF	Panel negative, WES pending
96	13	M	Yes	*KMT2C*	Kleefstra syndrome 2, MIM 617768	
97	11	M	Yes	*HMGCS2*	HMG-CoA synthase-2 deficiency, MIM 605911	
98	11	M	Yes	*KCNQ3*	Seizures, benign neonatal, 2, MIM 121201	
99	11	F	Yes	*SCN8A*	Developmental and epileptic encephalopathy 13, MIM 614558	
100	2	F	No	NA	ISS, DEE	Testing ordered, but not done (patient deceased)
101	11	M	Yes	*NALCN*	Hypotonia, infantile, with psychomotor retardation and characteristic facies 1, MIM 615419	
102	11	F	No	NA	GE, ID, ASD, GAD	Microarray negative
103	12	F	No	NA	CP	Testing ordered, but not done (lost to follow-up)
104	10	M	No	NA	ADHD, failure to thrive	Testing ordered, but not done (lost to follow-up)

105	10	F	Yes	*CNTNAP2*	Pitt-Hopkins syndrome	
106	10	F	Yes	*TSC2*	TSC	
107	5	F	Yes	*NPRL3*	Epilepsy, familial focal, with variable foci 3, MIM 617118	
108	2	F	Yes	*ATP1A3*	Developmental and epileptic encephalopathy 99, MIM 619606	
109	4	M	No	NA	ASD, ISS	Testing ordered, but not done (lost to follow-up)
110	2	F	Yes	*FGFR3*	Muenke syndrome, MIM 602849	
111	2	F	Yes	*FOXG1*	Rett syndrome, congenital variant, MIM 613454	
112	5	M	Yes	*DYNC1H1*	Cortical dysplasia, complex, with other brain malformations 13, MIM 614563	
113	2	M	Yes	*ADAR*	Aicardi-Goutieres syndrome 6, MIM 615010	
114	2	M	No	NA	CP	VUS *FOLR1*
115	2	M	Yes	*HMGA2*	Silver-Russell syndrome 5, MIM 618908	
116	5	F	No	NA	DEE	VUS *COL18A1*
117	2	M	Yes	del2q del15q	DEE	
118	5	F	Yes	*RARS2*	Pontocerebellar hypoplasia, type 6, MIM 611523	
119	2	M	Yes	*ARID1B*	Coffin-Siris syndrome 1, MIM 135900	
120	2	M	No	NA	ISS	Testing ordered, but not done (lost to follow-up)
121	2	M	No	NA	DEE	VUS *CAD FOXG1 PCLO PLCB1*
122	2	F	Yes	*TSC2*	TSC	
123	2	F	Yes	*CDKL5*	CDD	
124	2	M	No	NA	Neonatal seizures	VUS *DYNC1H1 PSAT1*
125	4	F	No	NA	ISS, CP	Panel and WES negative, WGS pending
126	5	M	Yes	*COL4A1*	*COL4A1*-related disorder, MIM 120130	
127	1.8	F	Yes	*SCN1A*	Dravet syndrome	
128	1.8	M	Yes	*CDKL5*	CDD	
129	2	F	No	NA	Neonatal seizures	VUS *ASAH1, DEPDC5, PEX6*
130	2	M	Yes	*NPRL3*	Epilepsy, familial focal, with variable foci 3, MIM 617118	
131	2	F	Yes	*NPRL3*	Epilepsy, familial focal, with variable foci 3, MIM 617118	

132	3	F	Yes	*NPC1*	Niemann-Pick disease, MIM 257220	
133	9	M	No	NA	FE	Single-gene testing for *TSC1/2* negative
134	9	M	No	NA	ASD, DD	WES negative
135	9	F	Yes	*COL4A1*	*COL4A1*-related disorder, MIM 120130	
136	9	F	Yes	*SYNE1*	Spinocerebellar ataxia, autosomal recessive 8, MIM 610743	
137	8	F	Yes	*POLG*	Mitochondrial DNA depletion syndrome 4A (Alpers type), MIM 203700	VUS *KCNJ10; POLG* het c.3708G>T; dup19q13.31q13.32
138	8	M	Yes	*FOXG1*	Rett syndrome, congenital variant, MIM 613454	
139	9	M	Yes	*KRAS*	Schimmelpenning-Feuerstein-Mims syndrome, somatic mosaic, MIM 163200	Somatic variant confirmed by biopsy
140	8	F	Yes	*GNAQ*	Sturge-Weber syndrome, somatic mosaic, MIM 185300	
141	13	F	Yes	*CHD2*	Developmental and epileptic encephalopathy 94, MIM 615369	
142	5	F	Yes	*FOXG1*	Rett syndrome, congenital variant, MIM 613454	
143	8	F	Yes	*ATP1A3*	Developmental and epileptic encephalopathy 99, MIM 619606	
144	9	M	No	NA	ISS, DEE	VUS *MBD5*
145	8	M	Yes	*DEAF1*	Neurodevelopmental disorder with hypotonia, impaired expressive language, and with or without seizures, MIM 617171	
146	8	F	Yes	21q22.3	Down syndrome, MIM 190685	and PIK3CA-related overgrowth disorder
147	8	F	Yes	*PDHA1*	Pyruvate dehydrogenase E1-alpha deficiency, MIM 312170	
148	8	M	Yes	*SCN1A*	GEFS+	
149	2	M	Yes	*ACOX1*	Peroxisomal acyl-CoA oxidase deficiency, MIM 264470	
150	8	F	Yes	*TSC2*	TSC	
151	8	M	Yes	*KCNB1*	Developmental and epileptic encephalopathy 26, MIM 616056	
152	8	M	No	NA	DEE, ASD	VUS *NRXN1, S1K1*
153	8	M	Yes	dup15q11-q13	dup15q11-q13 syndrome	
154	7	M	Yes	*DNMT3A*	Tatton-Brown-Rahman syndrome, MIM 615879	
155	7	M	No	NA	LGS	Panel negative, WES pending
156	7	M	Yes	*SPTAN1*	Developmental and epileptic encephalopathy 5, MIM 613477	
157	8	M	Yes	*TSC2*	TSC	

158	7	F	Yes	*SZT2*	Developmental and epileptic encephalopathy 18, MIM 615476	
159	8	F	Yes	4p partial trisomy, 22q partial monosomy syndrome	4p partial trisomy, 22q partial monosomy syndrome	
160	13	F	Yes	*SLC9A6*	Intellectual developmental disorder, X-linked syndromic, Christianson type, MIM 300243	
161	11	M	No	NA	LGS	VUS *IQSEC2*
162	7	M	Yes	*L1CAM*	Corpus callosum, partial agensis of, MIM 304100	
163	7	M	Yes	*MEF2C*	Neurodevelopmental disorder with hypotonia, stereotypic hand movements, and impaired language, MIM 613443	
164	7	M	No	NA	DEE, ASD, ISS	Panel negative, WES pending
165	7	M	Yes	del20p12.3-20p12.2	DEE, heterotaxy with cardiac defect	deletion including *BMP2, HAO1, PLCB1, PLCB4, LAMP5, PAK7*
166	7	F	Yes	*CDKL5*	CDD	
167	7	M	No	NA	DEE, ISS	Testing negative, not specified
168	6	M	Yes	*CDKL5*	CDD	
169	7	F	Yes	*SCN1A*	Dravet syndrome	
170	5	M	Yes	*FOXG1*	Rett syndrome, congenital variant, MIM 613454	
171	9	F	No	NA	CP, DD, GE, GAD, ASD	Testing ordered, but not done (lost to follow-up)
172	7	M	Yes	*NF1*	NF	
173	9	F	No	NA	Anti-NMDA-R encephalitis	Unclear case with positive family history
174	6	F	Yes	*PHOX2B*	Central hypoventilation syndrome, congenital, 1, with or without Hirschsprung disease, MIM 209880	
175	6	M	No	NA	DEE, ISS, DD, ASM	VUS *HNRNPU, RYR3, SATB2, SCN5A, SPATA5*
176	7	F	No	NA	DEE, ASD	VUS *NSD2, LAMB1*
177	6	F	No	NA	DEE	Testing ordered, but not done (lost to follow-up)
178	6	M	Yes	*SYN1*	Epilepsy, X-linked 1, with variable learning disabilities and behavior disorders, MIM 300491	
179	9	F	Yes	*TRIT1*	Combined oxidative phosphorylation deficiency 35, MIM 617873	
180	6	F	No	NA	Congenital heart disease	Evaluated for suspected Marfan syndrome
181	6	M	No	NA	Central hypoventilation syndrome	Single-gene testing (*PHOX2B*) negative
182	7	M	No	NA	BrS	Seizures and abnormal EEG likely incidental
183	6	F	No	NA	DD	Testing ordered, but not done (lost to follow-up)

184	6	F	Yes	*TSC*	TSC	
185	6	F	Yes	*SCN2A*	Developmental and epileptic encephalopathy 11, MIM 613721	
186	6	F	Yes	del20p, not specified	DEE	
187	6	F	No	NA	BFNE	VUS *CTSD*
188	6	M	Yes	*TBC1D24*	Developmental and epileptic encephalopathy 16, MIM 615338	
189	5	F	Yes	*COL4A1*	*COL4A1*-related disorder, MIM 120130	
190	5	M	No	NA	Glycogen storage disorder	Panel and WES negative, WGS pending
191	5	M	No	NA	ISS, DEE	VUS *SCN8A*, VUS 10q21.3
192	5	M	No	NA	ISS, DEE, ataxia, optic atrophy	Testing ordered, but not done (lost to follow-up)
193	5	F	Yes	*SCN2A*	Seizures, benign familial infantile, 3, MIM 607745	
194	5	M	No	NA	CP	Testing declined by patient
195	5	F	Yes	15q11.2	Angelman syndrome	
196	5	F	Yes	*CDKL5*	CDD	
197	5	F	No	NA	ASD, regression, lymphedema	VUS *BRWD3 SCN2A*
198	5	F	No	NA	DD	Testing negative, not specified
199	6	M	No	NA	DEE, congenital heart disease	Testing ordered, but not done (lost to follow-up)
200	5	F	No	NA	ISS, DEE	VUS *CACNA1A*
201	5	F	No	NA	ISS, DEE	VUS *SCN2A*
202	8	F	Yes	*CDKL5*	CDD	
203	5	F	Yes	*CDKL5*	CDD	
204	5	M	Yes	*ARX*	Developmental and epileptic encephalopathy 1, MIM 308350	
205	5	M	Yes	*STXBP1*	Developmental and epileptic encephalopathy 4, MIM 612164	
206	5	F	Yes	del16p13.3	Chromosome 16p13.3 deletion syndrome, MIM 610543	
207	4	F	Yes	*TSC2*	TSC	
208	4	M	No	NA	DEE, macrocephaly, failure to thrive, brain malformation	Microarray and single-gene testing negative
209	4	M	No	NA	DEE	VUS *GABRB2*
210	5	M	Yes	*HESX1*	Septooptic dysplasia, MIM 182230	

211	5	F	Yes	*KRAS*	Schimmelpenning-Feuerstein-Mims syndrome, somatic mosaic, MIM 163200	Somatic variant confirmed by biopsy
212	5	M	No	NA	ISS, DEE	Panel negative, WES pending
213	4	M	Yes	*CTNNB1*	Neurodevelopmental disorder with spastic diplegia and visual defects, MIM 615075	
214	6	M	Yes	*XXY*	Klinefelter syndrome	
215	4	M	Yes	del16p11.2	Chromosome 16p11.2 deletion syndrome, MIM 611913	
216	6	M	Yes	15q11.2	Angelman syndrome	
217	4	F	Yes	*CDKL5*	CDD	
218	4	M	Yes	*SLC13A5*	Developmental and epileptic encephalopathy 25, with amelogenesis imperfecta, MIM 615905	
219	8	M	No	NA	ASD, DD	VUS *CHRNA4*
220	4	M	Yes	*FOXG1*	Rett syndrome, congenital variant, MIM 613454	
221	4	F	No	NA	DEE, complex malformations, congenital heart disorder	VUS *PIK3R2*
222	4	F	Yes	*KIF11*	Microcephaly with or without chorioretinopathy, lymphedema, or mental retardation, MIM 152950	
223	4	F	Yes	15q11-q13	Prader-Willi syndrome, MIM 176270	
224	2	F	Yes	*TBX1*	DiGeorge syndrome, MIM 188400	
225	5	F	No	NA	DEE	VUS *FLNA*
226	4	M	Yes	*SCN1A*	Dravet syndrome	
227	4	M	No	NA	DEE	VUS *ADAR, NEDD4L, NPRL3, RFT1*
228	4	F	Yes	*DDX3X*	Intellectual developmental disorder, X-linked syndromic, Snijders Blok type, MIM 300958	
229	4	F	Yes	*SCN2A*	Seizures, benign familial infantile, 3, MIM 607745	
230	5	F	No	NA	DEE, microcephaly	VUS *CACNA1H, MTOR*
231	4	F	No	NA	DEE, ISS, agenesis of corpus callosum	Testing declined by patient
232	5	M	Yes	*NSD1*	Sotos syndrome	
233	4	F	Yes	*STXBP1*	Developmental and epileptic encephalopathy 4, MIM 612164	VUS *PNPO, RELN*
234	7	F	Yes	*FRRS1L*	Developmental and epileptic encephalopathy 37, MIM 616981	
235	3	M	Yes	*ADAR*	Aicardi-Goutieres syndrome 6, MIM 615010	And congenital heart disease
236	8	M	No	NA	DEE, schizencephaly	mtDNA negative, further testing pending
237	6	M	No	NA	DEE	VUS *DDX3X, RELN, MT-RNR2*

238	4	F	No	NA	ISS, DEE	Panel and WES negative, WGS pending
239	3	M	No	NA	FE, myoclonic epilepsy	VUS *SCN3A*
240	3	M	Yes	*KMT2C*	Kleefstra syndrome 2, MIM 617768	
241	3	F	Yes	*DOCK6*	Adams-Oliver syndrome, MIM 2614219	
242	3	M	No	NA	Arthrogryposis multiplex congenita	Multiple VUS
243	4	M	Yes	*HSD17B10*	HSD10 mitochondrial disease, MIM 300438	
244	3	M	Yes	del20q13.33	20q13.33 microdeletion syndrome	
245	5	M	No	NA	LGS	VUS *CASK*
246	3	M	Yes	*L1CAM*	Corpus callosum, partial agensis of, MIM 304100	
247	4	M	Yes	*TP53*	Li-Fraumeni syndrome, MIM 151623	
248	3	F	Yes	*NPRL3*	Epilepsy, familial focal, with variable foci 3, MIM 617118	
249	3	M	Yes	*CDKL5*	CDD	

Supplementary Table 3. Results of manual chart review. Abbreviations: ADNLFE – autosomal-dominant nocturnal frontal lobe epilepsy; ASD – autism spectrum disorder; ADHD – attention-deficit hyperactivity disorder; BFNE – benign familial neonatal seizures; BrS – Brugada syndrome; CDD – *CDKL5* deficiency disorder; CP – cerebral palsy; DD – developmental delay; DEE – developmental and epileptic encephalopathy; FraX – fragile X syndrome; FE – focal epilepsy; GAD – generalized anxiety disorder; GE – generalized epilepsy; GEFS+ – genetic epilepsy with febrile seizures plus; GLUT1-DS – Glucose Transporter Type 1 Deficiency Syndrome; HME – hemimegalencephaly; HSP – hereditary spastic paraplegia; ID – intellectual disability; ISS – Infantile Spasms Syndrome; LGS – Lennox-Gastaut syndrome; MIM – Mendelian Inheritance of Man; NEDHSB – neurodevelopmental disorder with hearing loss, seizures, and brain abnormalities; NF – neurofibromatosis; PDD – pervasive developmental delay; PDE – pyridoxine-dependent epilepsy; SCZ – schizophrenia; TSC – tuberous sclerosis complex; VUS – variant of unknown significance; WES – whole-exome sequencing;

Supplementary Figure 3: Validity analysis of age-dependent healthcare utilization

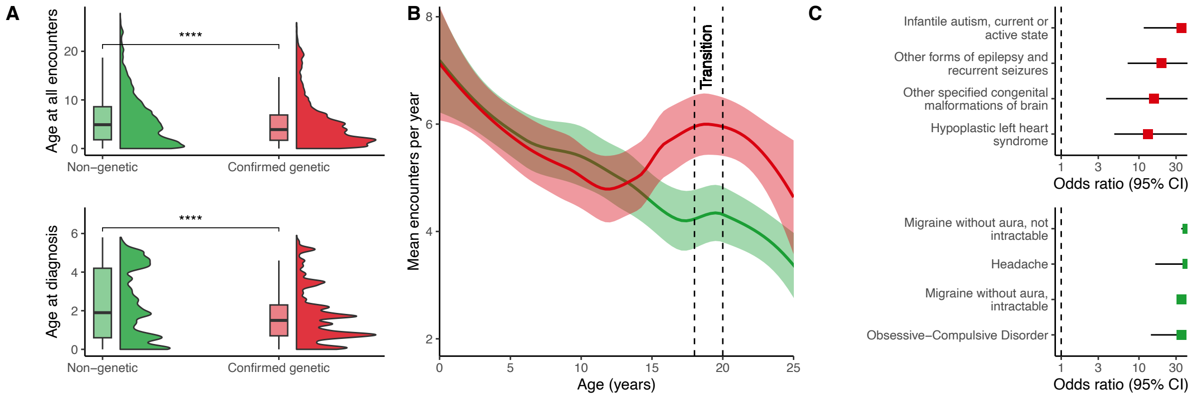

Supplementary Figure 3. Validity analysis of the age distribution and encounter distribution for the validation cohort. A: Individuals with confirmed genetic epilepsy are younger at all encounters with the healthcare system (mean age 5.23 SD 4.92 years vs. 5.96 SD 5.17, two-sided t-test, ****$p < 0.0001$) and are younger when they are first diagnosed with epilepsy (age at ICD-10 G40.-, mean age 1.77 SD 1.41 years vs. 2.26 SD 1.77, two-sided t-test, ****$p < 0.0001$) when compared to matched controls who received negative genetic testing. **B:** Individuals with confirmed genetic epilepsy have more complex encounters (mean concepts per encounter 8.14 (SD = 2.74) vs. 6.07 (SD = 2.66), two-sided t-test, $p = 3.13\text{x}10^{-17}$) and more annual encounters during transition, 6.74 (SD = 2.96) vs. 3.90 (SD = 2.53), two-sided t-test, $p = 1.84\text{x}10^{-5}$. The plot shows the mean number of annual encounters per year for each group. The line corresponds to the smooth conditional mean, with the shaded area being the standard error of the mean. The dashed lines mark the largest relative difference in annual encounter frequency, the transition period from pediatric to adult care (ages 18 – 20 years). **C:** Encounters for individuals with confirmed genetic epilepsy during the transition period are enriched for severe comorbidities including autism and congenital malformations of the brain, while individuals with non-genetic epilepsy are seen for other, possibly unrelated issues. Forest plot of UMLS concept enrichment during the transition period compared to before the transition period, sorted by highest odds ratio and shown separately for each group by color.

Supplementary Figure 4: Analysis of healthcare-related costs

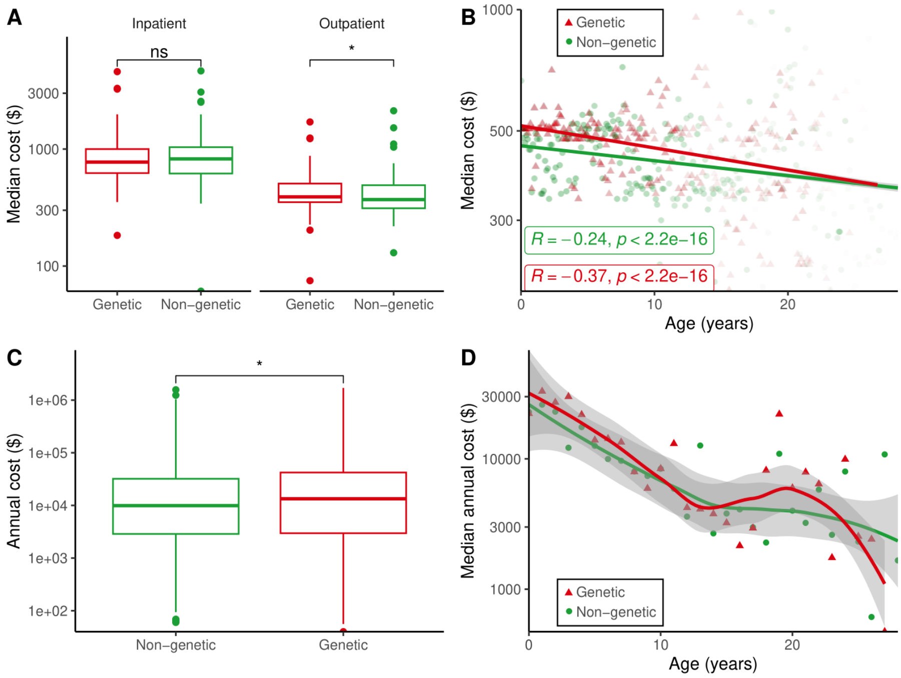

Supplementary Figure 4. Cost-based analysis of healthcare utilization in the validation cohort. A: Individuals with confirmed genetic epilepsy had higher costs per outpatient encounter (median cost before insurance adjustment 390 USD, IQR = 155 USD, vs. 370 USD, IQR = 179 USD; two-sample Wilcoxon test, p = 0.0215), but not per inpatient encounter. Cost was extracted from an internal claims database, reflecting the amount charged before insurance adjustment. **B:** The higher cost per encounter was consistent across the age range, and healthcare-related costs decreased with increasing age. Correlation between median encounter cost and age for each group are shown as Pearson's correlation coefficient. **C:** Annual healthcare-related costs were higher for individuals with genetic epilepsy compared to those with non-genetic epilepsy (13,371 USD, IQR = 39,186 USD, vs. 9,898 USD, IQR = 29,161 USD; two-sample Wilcoxon test, p = 0.0178). This annual sum more accurately reflects that the fact that individuals with genetic epilepsy also have more frequent encounters with the healthcare system, thus magnifying the financial burden. **D**: Median annual healthcare-related cost decreases with age but increases around the age of transition from paediatric to adult care in individuals with genetic epilepsy.

Supplementary Figure 5: Validity analysis of known and novel clinical characteristics

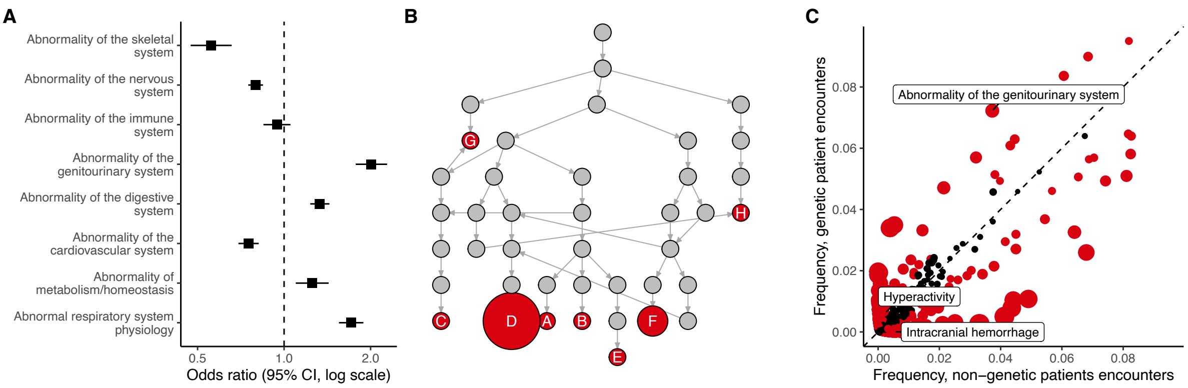

Supplementary Figure 5. Validity analysis confirms the cross-sectional analysis of clinical features associated with a likely genetic etiology. A: Individuals with genetic epilepsy are unexpectedly enriched for abnormalities of the genitourinary system (HP:0000119; OR 2.01, 95% CI: 1.78 – 2.28, $p_{adj} = 3.27 \times 10^{-27}$). Forest plot of system-level HPO terms that are children of phenotypic abnormality (HP:0000118). **B:** Reingold-Tilford tree visualization of the subgraph rooted at abnormality of the genitourinary system. Nodes shown in red are terms that are independently significantly associated with individuals with likely genetic epilepsy and are labelled by the term they represent: A - ectopic kidney (HP:0000086), B - polycystic kidney dysplasia (HP:0000113), C - chronic kidney disease (HP:0003744), D - penile hypospadias (HP:0003244), E - hydronephrosis (HP:0000126), F - cryptorchidism (HP:0000028), G - abnormality of urine homeostasis (HP:0003110), H - urinary incontinence (HP:0000020). **C:** Relative frequency of HPO terms in encounters for individuals with confirmed genetic epilepsy versus those with non-genetic epilepsy. Each dot corresponds to a single term and is coloured red if significant. Encounters of those healthcare system for individuals with genetic epilepsy are enriched for behavioural abnormalities such as hyperactivity (HP:0000752, OR 2.01, 95% CI: 1.64 – 4.31, $p_{adj} = 0.016$) or attention deficit (HP:0007018; OR 2.62, 95% CI: 1.64 – 4.30, $p_{adj} = 0.017$). We observed no associations with simple febrile seizures (HP:0011171), but an enrichment for complex febrile seizures (HP:0011172; OR 6.94, 95% CI: 2.45 – 27.05, $p_{adj} = 0.011$). Meanwhile, causes of symptomatic epilepsy such as cerebral haemorrhage were strongly depleted (HP:0001342, OR 0.02, 95% CI: 0.01 – 0.09, $p_{adj} = 1.67 \times 10^{-14}$).

Supplementary Figure 6: Validity analysis of longitudinal clinical characteristics and treatment patterns

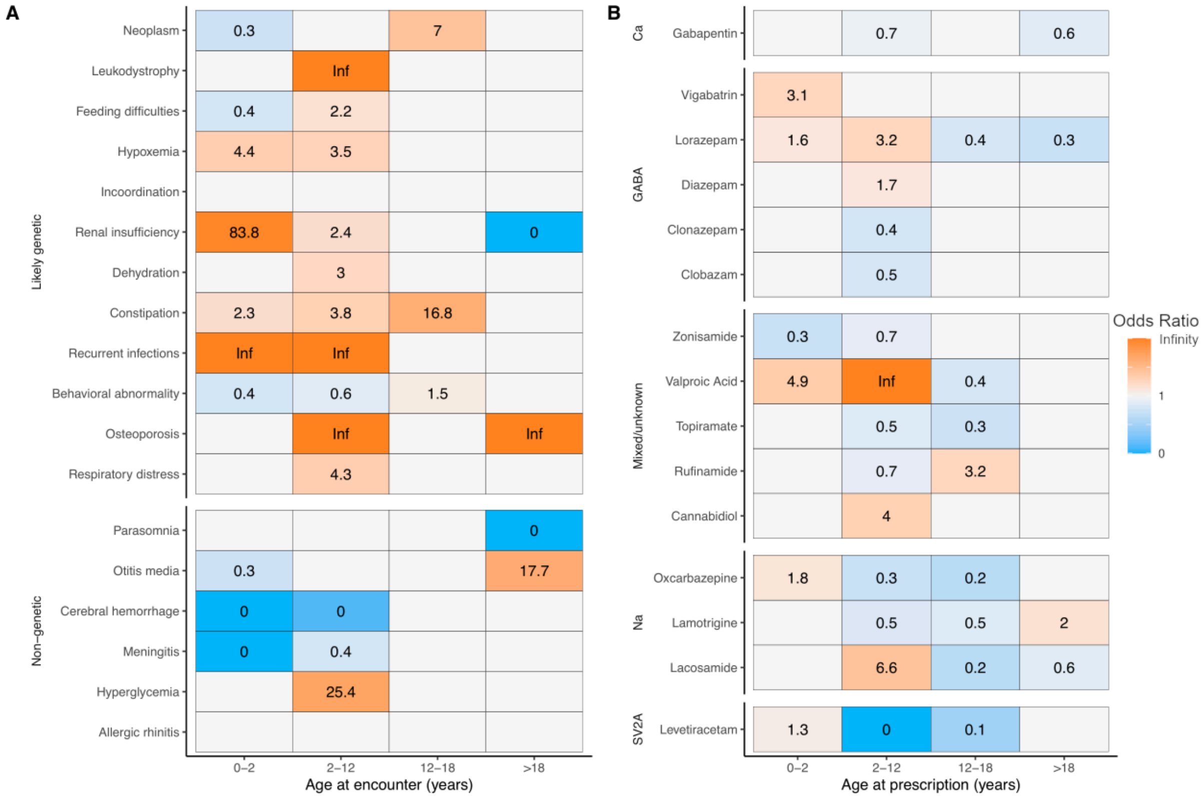

Supplementary Figure 6. Validity analysis of the longitudinal analysis of clinical features associated with confirmed genetic epilepsy. A: Heatmap of clinical features over the age ranges, binned by neonatal/infantile (0-2 years), childhood (2-12 years), juvenile (12-18 years), and adulthood (>18 years). Relative enrichment (odds ratio) of features between individuals with likely genetic epilepsy and those with non-genetic epilepsy is shown as labels. Blank tiles correspond to non-significant associations. Terms were grouped via hierarchical clustering of similar trajectories. **B:** Heatmap of anti-seizure medication (ASMs) prescription patterns, grouped by putative main mechanism of action. ASMs are shown if they had any significant group-level associations and were prescribed to at least 1% of the study cohort.

Supplementary References

1 Boßelmann CM, Hedrich UBS, Lerche H, Pfeifer N. Predicting functional effects of ion channel variants using new phenotypic machine learning methods. *PLOS Comput Biol* 2023; **19**: e1010959.
